# Multi-cellular network model predicts alterations in glomerular endothelial structure in diabetic kidney disease

**DOI:** 10.1371/journal.pcbi.1013598

**Published:** 2025-10-23

**Authors:** Krutika Patidar, Ashlee N. Ford Versypt

**Affiliations:** 1 Department of Chemical and Biological Engineering, University at Buffalo, The State University of New York, Buffalo, New York, United States of America; 2 Department of Biomedical Engineering, University at Buffalo, The State University of New York, Buffalo, New York, United States of America; 3 Institute for Artificial Intelligence and Data Science, University at Buffalo, The State University of New York, Buffalo, New York, United States of America; 4 Department of Pharmaceutical Sciences, University at Buffalo, The State University of New York, Buffalo, New York, United States of America; University of Virginia, UNITED STATES OF AMERICA

## Abstract

The progression of diabetic kidney disease is often characterized by early dysfunction of glomerular endothelial cells, including alterations in fenestration size and number linked to impaired glomerular filtration. However, the cellular mechanisms regulating fenestrations in glomerular endothelial cells remain poorly understood due to limitations in existing *in vitro* models, challenges in imaging small fenestrations *in vivo*, and inconsistencies between *in vitro* and *in vivo* findings. This study used a logic-based protein-protein interaction network model with normalized Hill functions for dynamics to explore how glucose-mediated signaling dysregulation impacts fenestration dynamics in glomerular endothelial cells. Key drivers of fenestration loss and size changes were identified by incorporating signaling pathways related to actin remodeling, myosin light chain kinase, Rho-associated kinase, calcium, and VEGF and its receptors. The model predicted how hyperglycemia in diabetic mice leads to significant fenestration loss and increased size of fenestrations. Glycemic control in the pre-diabetic stage mitigated signaling dysregulation but was less effective as diabetic kidney disease developed and progressed. The model suggested alternative disease intervention strategies to maintain the integrity of the fenestration structure, such as targeting Rho-associated kinase, VEGF-A, NFκB, and actin stress fibers.

## Author summary

Diabetic kidney disease is a serious complication of diabetes that affects approximately 20–40% of diabetic patients and can lead to kidney failure. Early diagnosis and treatment of diabetic kidney disease are critical in slowing the progression of the disease and preventing kidney failure. The progression of diabetic kidney disease is often characterized by structural changes in glomerular endothelial cells, which are important cells in the kidney zones responsible for filtration. In this work, we expanded our previous model of chemical interactions in glomerular endothelial cells that focused on cell culture data from experiments outside an organism. Here, the model is adapted to data from mice. Using a protein-protein interaction network, we computationally examined key pathways that relate hyperglycemia in diabetic mice to significant structural changes in glomerular endothelial cells. The analysis identified that targeting specific pathways and proteins could maintain structural integrity when glucose control alone is insufficient. The proposed disease targets and biomarkers are closely correlated with structural changes in glomerular endothelial cells and could support improved kidney function and disease management.

## Introduction

The kidney is a highly vascularized organ that includes different populations of endothelial cells (ECs) with specialized structures and functions [1]. ECs in the kidney microvasculature regulate blood flow, coagulation, inflammation, and vascular permeability [1,2]. In the functional unit of the kidney, glomerular endothelial cells (GECs) are highly specialized ECs that contribute to the structural and functional integrity of the glomerular filtration barrier in each nephron and support other glomerular cells, such as podocytes and mesangial cells, and the glomerular basement membrane [3,4].

The structure of GECs consists of transcellular holes, known as fenestrations, and a rich surface of glycocalyx that together contribute to the size- and charge-selective properties of the filtration barrier [1,5–8]. The endothelial glycocalyx lines the luminal side of the GECs and is also present within the fenestrations [7,9]. The functional significance of fenestrations is to provide selective passage of proteins, fluid, and small solutes across the GEC barrier without the need for endocytosis or receptor-mediated mechanisms [7,10]. The fenestrated endothelium regulates the glomerular filtration rate and permeability [5,9,10]. Mature GEC fenestrations are generally open and non-diaphragmed, located in the peripheral cytoplasm, and arranged in non-raft sieve plates [5,9,11,12]. The fenestrations are supported by a fenestrae-associated cytoskeletal ring [13,14], wherein structural proteins like spectrin and filamin crosslink with the actin cytoskeleton, contributing to fenestration formation, membrane integrity, cell-cell interactions, and shape changes [2,13–15]. Although their protein composition remains mostly unknown, studies have determined the structural composition of fenestrations [13,16,17]. In addition to structural proteins, vascular endothelial growth factor (VEGF), endothelin-1, and tumor necrosis factor (TNF)-α are among other agents that modulate endothelial fenestration structure and vascular permeability [2,12]. Shear stress also regulates the production of vasoactive mediators, such as nitric oxide (NO) and endothelin-1, and regulates fenestration structure and vascular tone [18].

GECs are susceptible to injury and dysfunction in kidney diseases. Alterations in the size and density of GEC fenestrations are associated with the disruption in glomerular filtration and progression of diabetic kidney disease (DKD) [9,10,19]. DKD is a microvascular dysfunction in the kidneys and is reported in 20–50% of diabetic patients [20]. DKD is the leading cause of end-stage renal failure and is associated with significantly increased comorbidities and mortality [10,20]. GEC activation and dysfunction are considered early signs of DKD development and progression [6,21]. GEC activation results from dysregulated paracrine and autocrine signals in these cells triggered by high glucose, inflammation, or injury [6] and progresses to endothelial dysfunction involving changes in structure and function. In our study, we consider the early stage of DKD to be 6–10 weeks in mice, i.e., after sustained hyperglycemia and before significant histological changes in cell structure are observed around 10–12 weeks as in prior studies [10,22].

Some recent studies have focused on understanding dysfunction and injury in endothelial cells in a diseased state. Accumulated evidence associates pathways and signaling motifs with actin cytoskeletal rearrangement and, ultimately, morphological changes in fenestrations in liver sinusoidal endothelial cells (LSECs), which are structurally similar to GECs [9]. VEGF, NO, and calcium are linked to the regulation of myosin light chain kinases (MLCK), Rho, and Rho-associated protein kinase (Rock) [2,13,14,23–25]. Calcium-dependent inactivation of MLCK and Rho/Rock activation reduces endothelial porosity in LSECs and, in some cases, increases the diameter of fenestrations [13,14,26]. Although most previous studies were performed on fenestrated LSECs, some recent studies focused on fenestrated GECs [2,10]. Despite some progress toward a mechanistic understanding of markers and pathways associated with GEC dysfunction, research gaps remain in understanding the relationship between signaling molecules and mechanical cues in GECs that cause structural deformation. Using the information about pathways in other fenestrated ECs is a promising strategy for understanding these pathways’ potential impacts on the structural or functional stability of GECs before they can be validated experimentally in GECs.

Computational network models have been shown to be useful in mechanistically linking signaling cues to cellular dysfunction and activation. Previously, we [27] and others [28–30] used computational modeling to demonstrate the intracellular signaling and intercellular cross talk among pro-inflammatory mediators, pro-angiogenic factors, immune cells, and endothelial cells. We previously developed a logic-based ordinary differential equations (LBODEs) model to predict the effects of high glucose and inflammation on macrophage and GEC activation and signaling dysregulation observed *in vitro* associated with early-stage DKD [27]. Others have also used complex network models to study different macrophage phenotypes in response to mixed pro- and anti-inflammatory stimuli [30,31]. Several large-scale logic-gated network models have effectively determined signaling components and network topology that regulate cell phenotype, function, and structure in other tissues [32,33].

In this study, we used an LBODEs model of protein-protein interaction to study the development and progression of DKD. Here, we modified and extended our previously developed LBODEs model [27] to include essential proteins and interactions that potentially modulate fenestration density and size in GECs *in vivo*. We calibrated the influential parameters in the extended LBODEs model using published experimental data of observed changes in fenestration size and number in diabetic mice. Using this extended LBODEs model, we analyzed network motifs and dynamics under varying glucose stimuli and perturbed protein activity. We identified potential strategies for therapeutic interventions to reduce fenestration loss across early to advanced stages of DKD.

## Methods

### Network assembly

**Fig 1:**
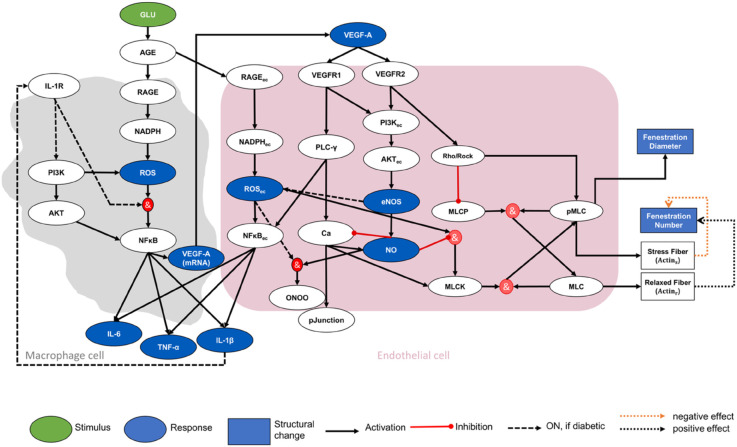
Multi-cellular protein interaction network of in vivo cross talk between macrophages and glomerular endothelial cells. The protein interaction network between macrophages (gray area) and glomerular endothelial cells (pink area) is stimulated with static or dynamic glucose. The green oval is the input node, the blue ovals are the output nodes, and the white ovals are the regulatory nodes. Structural changes in glomerular endothelial cells are shown as blue squares. The black solid arrows are activating interactions, the red edges with dots at one end are inhibiting interactions, and the gray dashed arrows are interactions active for diabetic subjects. Open dotted arrows in orange and black represent negative and positive effects on fenestration size, respectively. Red circles indicate logic *AND* gates. An *OR* logic rule connects two or more edges to a subsequent node throughout the network unless indicated otherwise by an *AND* logic gate. The subscript “ec” denotes an intracellular species expressed in endothelial cells. IL-6, TNF-α, IL-1β, and VEGF-A are protein levels expressed in the extracellular space. ROS, ROSec, VEGF-A (mRNA), and NO are expressed within the cells. The pJunction node represents the phosphorylated junction protein levels. Stress fibers and relaxed fibers represent different forms of actin fibers. AGE: advanced glycation end products. AKT: serine/threonine-specific protein kinases. Ca: calcium. eNOS: endothelial nitric oxide synthase. IL: interleukin. MLC: myosin light chain. MLCK: myosin light chain kinase. MLCP: myosin light chain phosphatase. NADPH: nicotinamide adenine dinucleotide phosphate. NFκB: nuclear factor kappa B. NO: nitric oxide. ONOO: peroxynitrite. PI3K: phosphoinositide 3-kinases. PLC-γ: phospholipase C gamma. pMLC: phosphorylated myosin light chain. RAGE: receptor of advanced glycation end product. Rock: RhoA-associated kinase. ROS: reactive oxygen species. TLR: toll-like receptor. TNF-α: tumor necrosis factor-alpha. VEGF: vascular endothelial growth factor. VEGFR: vascular endothelial growth factor receptor. New nodes and interactions in the extended model compared to those in the previous model [27] are highlighted in Fig A in S1 Appendix.

We modified our previously developed protein signaling network (PSN) between macrophages and GECs [27] to include relevant pathways and proteins associated with changes in GEC fenestrations (Fig 1). The previous network model [27] was assembled and validated based on evidence from pathway databases, cell culture experiments, transcriptomic analyses, and single cell RNA sequencing data [29,34–53]; please see [27] for more details on the rationale behind the assembly of the previous network stimulated by glucose and by an inflammatory lipopolysaccharide signal *in vitro*.

Several experimentally determined effects were considered to extend the previous PSN [27] to the network in Fig 1. The new regulatory nodes and interactions are highlighted in Fig A in S1 Appendix and were derived from proteins and mechanisms proposed in published experiments and hypotheses with fenestrated LSECs [13,14,16,25,54] and GECs [2]. The new regulatory nodes (white ovals in Figs 1 and A in S1 Appendix) include IL-1R, Rho/RhoA-associated kinase (Rock), myosin light chain (MLC), MLC phosphatase (MLCP), MLC kinase (MLCK), phosphorylated myosin light chain (pMLC), stressed actin fibers (Actin_s_), relaxed actin fibers (Actin_r_), fenestration number, and fenestration diameter. Fig A in S1 Appendix also highlights the new interactions between existing and new nodes in the extended model of Fig 1. Abbreviations for all species are defined in Table A in S1 Appendix.

Unlike the previous PSN, which was stimulated by static glucose and lipopolysaccharide stimuli [27], the extended model is stimulated by a dynamic glucose concentration in diabetic mice and an endogenous inflammatory stimulus (IL-1β) indirectly regulated by glucose via the macrophage cell portion of the network. Glucose is the only independent stimulus in the extended network model (Fig 1) and is responsible for initiating a phenotypic switch in macrophages, activating GECs, and initiating downstream signaling dysregulation. IL-1β activates its receptor IL-1R on macrophages in the extended PSN. These are the only modifications to the macrophage portion of the extended PSN.

The modifications to the endothelial cell portion of the extended PSN (Figs 1 and A in S1 Appendix) were of two types: biochemical and structural. First, the biochemical modifications are described. VEGF, a pro-angiogenic factor, increases EC porosity and permeability in different cell types [2,14,55–58] and promotes maintenance of fenestrations via NO-dependent or NO-independent pathways. We linked VEGF-A, VEGF receptors, NO, ROSec, and calcium in the previous PSN with additional nodes in the network. VEGF receptor 2 (VEGFR2) mediates Rho/Rock activation, leading to defenestration in LSECs [2,13,57]. Treatment with reactive oxygen species or nitrogen species increases fenestration diameter and decreases fenestration number [14]. The calcium level, regulated by calcium membrane channels and pumps, causes a cascade of cellular mechanisms that drive local changes in the cytoskeleton and result in actin contraction [14,59]. The exact mechanism of action of NO on fenestration has not been shown. However, it has been demonstrated that eNOS-derived NO shows a positive effect on LSEC fenestration maintenance [57]. It was proposed that activation of the NO-dependent cGMP pathway reduces the activation of MLCK [14]. The local balances regulating the calcium, ROS, NO, and VEGF levels in different parts of the cell control the dynamics of fenestrated LSEC [14] and are included in the extended network. The activity of MLCK is increased by calcium and protein kinase C (PKC)-mediated phosphorylation [14]. The link between MLCK and calcium is considered in the extended network. Moreover, indirect inhibition of MLCK, either through a calcium-dependent or calcium-independent manner, reduced endothelial porosity and increased fenestration diameter in a few cases [26]. Indirect inhibition of MLCK via NO and ROS activation is included in the extended network. MLCP maintains the balance of phosphorylation or dephosphorylation of MLC. MLCK and MLCP, together, keep the balance between pMLC and MLC protein levels (Fig 1). The Rho/Rock pathway activates pMLC protein and inhibits MLCP protein [14,26]. pMLC protein increases contractile forces in the actin cytoskeleton structure, which we modeled as activation of stress fibers in the extended network (Fig 1). Other agents, such as PKA, PKG, and PKC not explicitly included in the extended network, may also activate pMLC; however, they are not as potent as the Rho/Rock pathway [14]. RhoA regulates the assembly of contractile actin bundles and actin filaments, and negative regulators of Rho/Rock have resulted in massive proteinuria and renal failure in mice [60]. A previous study in mouse podocytes also indicated that the actin cytoskeleton could be a potential target for stabilizing cellular morphological changes, proteinuria, and renal function [60]. The interplays between pathways related to MLC kinase and phosphatase, Rho/Rock, calcium, NO, VEGF, VEGFR, and ROS were prominently observed in fenestrated LSECs [14]; therefore, these are considered in the extended network.

For the structural modifications to the endothelial cell portion of the extended PSN (Figs 1 and A in S1 Appendix), we assumed that the overall fenestration number depends on the actin cytoskeleton structure, which is regulated by the proportion of stressed and relaxed actin fibers. The role of actin cytoskeleton as an essential structural and functional element that controls cell shape, cell motility, and adhesion has been demonstrated in different cell types [60,61]. Myosins convert ATP to create a mechanical force on actin, which creates tension in the actomyosin cytoskeleton necessary for various functions [14]. Under a changing extracellular environment, actin structures are disassembled and remodeled to maintain the structural and functional integrity. Thick actin stress fibers have been associated with elevated regions (raft regions) in endothelial cell monolayers. According to the sieve-raft hypothesis [12], these elevated regions have no fenestrations. It has been postulated that the regulation of fenestration size in LSECs is facilitated by the contraction or relaxation of the cytoskeleton surrounding the sieve plates [17,26]. Fenestrations exist within the mesh-like structure comprised of actin [62]. The presence of stressed actin fibers leads to the loss of fenestrations, whereas relaxed fibers promote fenestration formation. Thus, we consider both to determine the changes in the fenestration number. As the thick actin fibers around the mesh-like structure cause it to stretch, the fenestrations increase in size [26]. On the other hand, when actin fibers relax and tension around the mesh-like structure loosens, the fenestration size reduces. Size is considered by the variable fenestration diameter. In the extended network, fenestration diameter is directly linked to pMLC protein, which influences both stressed and relaxed actin fibers.

### Logic-based network model development

The LBODEs modeling technique combines ordinary differential equations (ODEs) that are continuous functions of time with qualitative logic-based Boolean up- or down-regulation (i.e., activation or inhibition) using normalized Hill functions (saturating sigmoidal terms) for the logic-based modeling portion [27,63]. The LBODEs framework enables predictions of network dynamics and is compatible with many analyses from nonlinear dynamics while requiring minimal knowledge of biochemical parameters [32,64]. The LBODEs model uses normalized-Hill functions to define normalized species activity generally between 0 and 1 [27], although we allowed dynamic glucose stimulus beyond this range on the normalized scale.

The LBODEs model parameters are categorized as reaction parameters—reaction weight (*W*_*j*_), Hill coefficient (*n*_*j*_), and half-effect ($EC_{50_{j}}$) for reactions *j*—and species parameters—maximum species activity ($y_{max_{i}}$), initial species value ($y_{0_{i}}$), and time constant ($\tau_{i}$) for species *i*. Reaction weight regulates the strength of each network interaction, half-effect determines the amount of input activity required to achieve maximum output activity, and time constant controls the time to activate or inhibit a network species. The species *i* and reactions *j* are listed in Tables B and C in S1 Appendix. More detailed information on the LBODEs model structure, equations, and parameters can be found in our earlier work [27].

Our previous model [27] characterized the early stages of DKD development by simulating protein dysregulation in macrophages and GECs occurring over a few hours to days. This model was thoroughly calibrated and validated against *in vitro* experimental datasets. The default parameters as defined by Kraeutler et al. [63] allowed a qualitative prediction using the previous LBODEs model. The previous model was assessed for structural identifiability and observability to minimize uncertainty due to non-identifiable parameters and address structural issues before model calibration [27,65,66]. Given the large number of model parameters in the previous network model, we identified the most sensitive parameters based on a global sensitivity analysis using Sobol’s variance-based method [67–69]. As information on the model parameters were not available in the literature, we estimated the most sensitive parameters using a multi-start nonlinear least squares optimization routine and quantified the uncertainty associated with the estimated parameter values and how this propagated to model prediction uncertainty [27].

Here, we considered the expanded network (Fig 1) and *in vivo* data and scenarios. The optimal parameters from the previous LBODE model were used in the extended model for the respective nodes. Due to limited information on model parameters for the additional nodes of the extended LBODEs model, we set these new parameters at default values (*W*_*j*_ = 1, *n*_*j*_ = 1.4, $EC_{50_{j}}=0.5$, $y_{0_{i}}=0$, $y_{max_{i}}=1$, $\tau_{i}=1$), as in multiple other publications using LBODEs [30,32,63,70–73]. The cellular and protein expression responses to the glucose stimuli are governed by biochemical reactions and interactions in the network model. These reactions allow the transfer of information within or between cells, which can happen at a range of timescales (seconds to hours). The time constant parameters in the LBODEs model modulate the early or late activation of proteins in the network. The previous LBODEs model [27] was simulated and fitted for a short timescale (48 hours) suitable for *in vitro* studies. In the extended LBODEs model, the time constant parameters for each protein come from a published timescale analysis [74]. The previous timescale analysis was performed for biochemical reactions similar to those seen in the extended network model, where time constant values were grouped by reaction type. The reaction types in the extended network are categorized into the following reaction types: ligand-receptor, transcription, translation, NF-κB activation, and signaling reactions. The time constants for the reaction types are as follows: those for ligand-receptors are 21 min = 0.35 h, those for transcription are 88 hours, that for translation is 1.13 hours, that for NF-κB activation is 3.3 min = 0.055 h, and those for all other signaling interactions are 1 hour. For species, IL-6, TNF-α, and IL-1β, the total times for transcription and translation reactions are combined and approximated as 90 hours. The species and reaction parameters are listed in Tables B and C in S1 Appendix.

The equations of the extended LBODEs model in Eqs (S1)-(S34) in S1 Appendix for the extended PSN (Fig 1) were generated automatically using Netflux, an open-source software package [63,64]. The information about model parameters and reaction rules in the extended PSN (Tables B and C in S1 Appendix) is sufficient to generate the LBODEs using Netflux. The use of Netflux graphical user interface is optional to set up the MATLAB scripts and basic model structure. After defining the model equations in MATLAB or via Netflux, MATLAB software has many advanced capabilities and was used to perform parameter estimation, sensitivity analyses, and post hoc analyses. Here, the LBODEs model was simulated in MATLAB after model modifications for the dynamic glucose stimulus and the structural aspects of the endothelial cell portion of the extended PSN, as discussed below.

### Dynamic glucose stimulus

**Fig 2:**
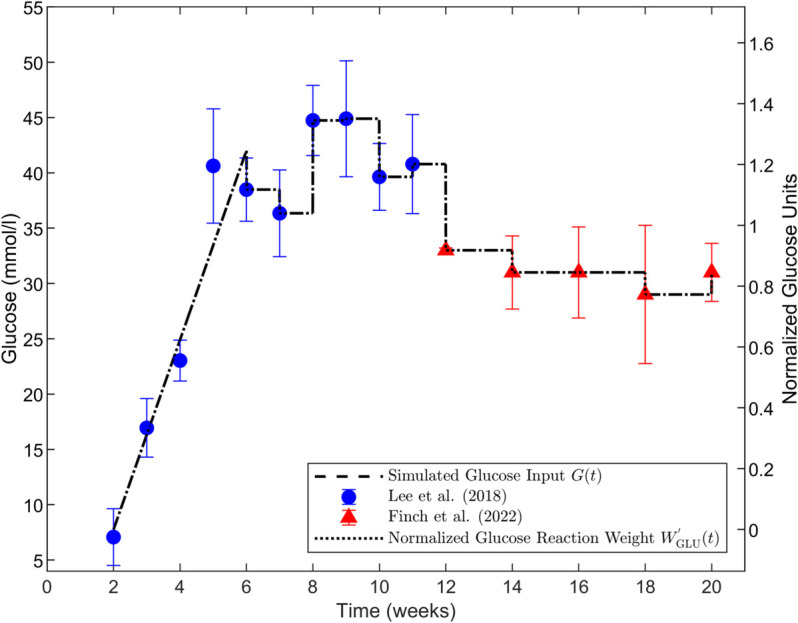
Simulated glucose input concentration profiles. Simulated glucose input concentration profiles (black dashed curve on left axis) over 20 weeks using the linear fit for 2–6 weeks from Eq (1) and piecewise constant values of glucose at the means of the observed data in diabetic mice for 6–20 weeks. Dimensional glucose values *G*(*t*) are shown on the left axis, and normalized values of glucose reaction weight $W_{GLU}^{\prime }(t)$ obtained using Eq (2) are shown on the right axis (black dotted curve). Glucose concentration data values are from Lee et al. [22] (blue circles) and Finch et al. [10] red triangles for male ob-/ob- mice. Data are shown as means ± standard deviations.

The LBODEs model is simulated between 336 and 3360 hours (2–20 weeks). The extended model is stimulated by dynamic glucose levels in leptin-deficient mice, denoted ob-/ob- or ob/ob, with BTBR background that develop severe type 2 diabetes [10,22]. This mouse strain models the later stages of DKD [75]. A representative data set of *in vivo* glucose dynamics in diabetic mice [22] is used as the stimulus for the development of diabetic hyperglycemia (Fig 2); glucose levels initially increase linearly from baseline and then fluctuate within a hyperglycemic range. We considered glucose concentration as a linear function Eq (1) and fitted it to data [22] for 336–1008 hours (2–6 weeks):

G(t)=0.051t−9.38,336 h≤t≤1008 h (1)

where *G*(*t*) is glucose concentration at time *t* in hours.

To mimic natural glucose dynamic variations without having fine details of feeding and metabolism, for *t* > 6 weeks, glucose is set to observed glucose concentrations in diabetic mice [10,22] using step changes at each time point. Glucose values are held constant during each time interval between the measured data standard deviations (Fig 2) at time points corresponding to published experimental measurement times: weekly until 11 weeks [22] and biweekly during 12–20 weeks [10]. Fig 2 shows the glucose data and simulated levels following Eq (1) for 2≤t<6 weeks and a piecewise constant function at the mean values of the data in each measurement time interval for t≥6 weeks. Data are shown as means ± standard deviations.

Glucose input to the LBODEs model must be a normalized value on the order of 1. Therefore, a variable reaction weight ($W_{GLU}^{\prime }(t)$) is calculated from glucose concentration at each time by normalizing *G*(*t*) between the minimum value from Eq (1) at *t* = 2 weeks and the maximum value (mean + standard deviation) from the data in the 12–20 weeks interval:

$$W_{GLU}^{\prime }(t)=\frac{G(t)-min(G(t))}{max(G(t))-min(G(t))}$$ (2)

where min(G(t)) and max(G(t)) are the minimum and maximum concentrations of observed glucose, respectively. Reported data by Finch et al. [10] were primarily used for calibration of the fenestration structural dynamics portion of the model. Therefore, we also calibrated the glucose normalization to this data set. Due to a lack of reported data for glucose between 2 and 12 weeks in [10], min(G(t)) is set to the predicted glucose concentration at 2 weeks computed using Eq (1). max(G(t)) is set to the maximum value of the upper bound of observed glucose from Finch et al. [10]. Therefore, a normalized glucose activity of 1 corresponds to the maximum mean + standard deviation of glucose concentration in mice reported in Finch et al. [10]. Note that this allows some normalized values from other data sets (specifically, the Lee et al. [22] data) to have values greater than 1. The right axis of Fig 2 shows the results of the normalization using Eq (2).

Normalized glucose activity (GLU) is used as the input to the extended LBODEs network and is calculated as

$$\frac{dGLU}{dt}=\frac{y_{max_{GLU}}W_{GLU}^{\prime }(t)-GLU}{\tau_{GLU}}$$ (3)

where $y_{max_{GLU}}$ is the maximal glucose activity and is set to 1, $W_{GLU}^{\prime }(t)$ is the dynamic reaction weight for glucose from Eq (2), and $\tau_{GLU}$ is the time constant for glucose and is set to 1. The GLU result from Eq (3) based on the *G*(*t*) in Fig 2 is shown in the first panel of Fig B in S1 Appendix.

### Glucose variability

**Fig 3:**
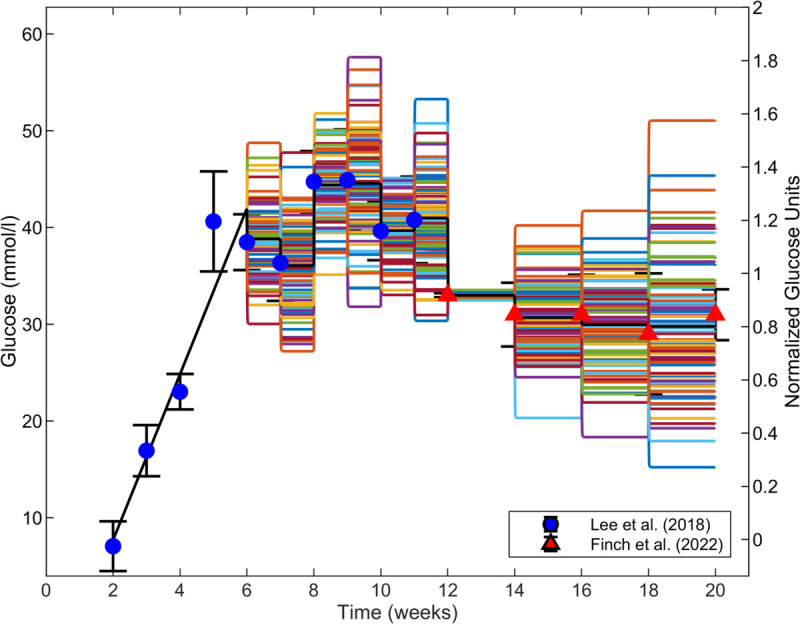
Simulated glucose input concentration profiles (solid colorful lines) for a virtual mouse population (*n* = 100) to represent time-dependent inter-subject glucose variability. Glucose was sampled *n* times in each time interval for 6–20 weeks by drawing from a normal distribution with mean and standard deviation from the data at the start of the corresponding time interval. The mean of the 100 profiles is shown as a solid black line. Glucose concentration data values are from Lee et al. [22] (blue circles) and Finch et al. [10] (red triangles). Data are shown as means ± standard deviations.

We simulated inter-subject variability of glucose concentration in the mouse population based on reported variability in previous studies [10,22]. We created an *in silico* virtual mouse population (*n* = 100) that varied in their input glucose concentration dynamics. As in Fig 2, we used the linear values following Eq (1) for 2≤t<6 weeks and piecewise constant functions at data-informed values in each measurement time interval for t≥6 weeks. The difference is that in Fig 2, we used the data-informed values as the means from the data in each interval. For inter-subject glucose variability, we sampled those values from distributions informed by the data. Specifically, we constructed the piecewise constant functions for the virtual mouse population for t≥6 weeks by drawing 100 independent samples from a normal distribution using the normrnd function in MATLAB with the mean and standard deviation corresponding to the data at the start of each time interval. Another 100 independent samples were drawn for the next time interval, which had its own mean and standard deviation, and this process proceeded until 20 weeks. Each virtual mouse got an arbitrary combination of one glucose sample per time interval for its piecewise constant function. We ensured that our glucose samples were the same 100 trajectories each time we reran the code by fixing the seed for the random number generator. The resulting 100 sampled glucose trajectories and the mean of these trajectories are shown in Fig 3. The visualization in Fig 3 has limitations in showing all 100 trajectories without obscuring frequent samples. As an alternative visualization, we have also provided histograms of the sampled GLU distributions in each time interval for 6–20 weeks (first column of Fig C in S1 Appendix).

### Dynamic changes in fenestration structure

We simulated the changes in fenestration number and diameter using a published *in vivo* study of GEC dysfunction in the advanced stage of DKD [10]. In our model, the fenestration number depends on the total activity of stressed and relaxed actin fibers. Relaxed actin fibers promote the formation of fenestrations at a rate $k_{form}$, and stressed actin fibers reduce fenestrations in GECs at a rate $k_{loss}$. Eq (4) defines the rate of change in fenestration number ($y_{Number}$) over time:

$$\frac{dy_{Number}}{dt}=k_{form}Actin_{r}|y_{Number_{ss1}}-y_{Number}{|}^{n_{f}}-k_{loss}Actin_{s}|y_{Number_{ss2}}-y_{Number}{|}^{n_{f}}$$ (4)

where $k_{form}$ and $k_{loss}$ are the rates of formation and loss of fenestrations, respectively. $y_{Number_{ss1}}$ and $y_{Number_{ss2}}$ are the numbers of fenestrations at steady state for healthy control and diabetic mice, respectively. *n*_*f*_ is the shape factor. $Actin_{s}$ and $Actin_{r}$ are stressed and relaxed states of actin fiber activity from Eqs (S1) and (S29) in S1 Appendix, respectively.

The predicted fenestration number was compared with the measured fenestration density in GECs in mice, where density is defined as the total number of fenestrations per unit length (pm) of the peripheral cytoplasm. The first term in Eq (4) defines a nonlinear increase in fenestration number compared to healthy mice. The second term in Eq (4) defines a nonlinear decrease in fenestration number compared to baseline values in healthy mice.

The phosphorylation of MLC protein (pMLC) in Eq (S32) in S1 Appendix is assumed to be the source of stress that increases fenestration diameter at a rate *k*_*s*_ Eq (5). This assumption is based on previous experimentation in fenestrated LSECs [14,26]. Eq (5) defines the rate of change in fenestration diameter ($y_{Diameter}$) over time:

$$\frac{dy_{Diameter}}{dt}=k_{s}(pMLC-pMLC_{0}{)}^{n_{f}}-k_{d}(y_{Diameter}-y_{Diameter_{0}})$$ (5)

where *k*_*s*_ and *k*_*d*_ are the rates of increase in diameter due to phosphorylation of MLC protein (units of nm/hr) and restoration of diameter (units of 1/hr), respectively. $pMLC_{0}$ and $y_{Diameter_{0}}$ are the initial values of normalized pMLC protein and diameter at baseline in control subjects or healthy mice. The increased fenestration diameter is restored via an unknown restoring force at a rate of *k*_*d*_.

### Parameter estimation

For parameters not used at default values, we calibrated the LBODEs model in Eqs (3)-(5) and Eqs (S1)-(S34) in S1 Appendix to *in vivo* data for the glucose dynamics and changes in fenestration structures for 12–20 weeks. We used the single mean glucose trajectory *G*(*t*) (Fig 2) as the input. We performed a multi-start nonlinear least squares parameter estimation to estimate unknown parameter values in Eqs (4) and (5). We used Latin hypercube samples to generate 25 initial sets of parameter from specified ranges for each parameter. $k_{form}$, $y_{Number_{ss1}}$, and *n*_*f*_ were sampled in the ranges of [0.1,4] 1/hr, [6,8], and [2,5], respectively. $k_{loss}$ and $y_{Number_{ss2}}$ were sampled in the ranges of [1,5] 1/hr and [3,5], respectively. *k*_*s*_, *k*_*d*_, and $\tau_{pMLC}$ were sampled in the ranges of [45,75] nm/hr, [1,4] 1/hr, and [400,600] hr, respectively. The parameter estimation was performed using fmincon in MATLAB. The optimization objective function was to minimize the sum of squared error (SSE) between the model predictions and the data. The optimization was repeated for the 25 sets of initial guesses of the parameters to account for the local minimization algorithm in fmincon. The best-fit parameter values were those that yielded the lowest SSE among the results from the 25 multi-start calls to the optimization algorithm.

Rather than estimating all parameters for Eqs (4) and (5) simultaneously, we partitioned the problem into multiple steps based on the available data. In the first step in the parameter estimation process, only the first term in Eq (4) was considered, and three parameters ($k_{form}$, $y_{Number_{ss1}}$, and *n*_*f*_) were fit to fenestration density at 6, 10, 15, and 20 weeks in healthy mice [10]. Some changes were made to the disease model to simulate fenestration formation in healthy cases. In healthy cases, we assumed a balance between relaxed and stressed actin fiber activity. To observe balanced actin fiber activity, we switched to an activation interaction between Rho/Rock and MLCP to promote the activation of relaxed actin fibers. We estimated $k_{form}$, $y_{Number_{ss1}}$, and *n*_*f*_ using this methodology.

In the second step, we considered the disease model (Fig 1) and both terms in Eq (4). In diseased cases, the model promotes an imbalanced expression of relaxed and stressed fibers. The relaxed actin fibers are inactive in the diseased model mainly due to Rock-mediated MLCP inhibition. Parameters $k_{loss}$ and $y_{Number_{ss2}}$ in Eq (4) that define the decrease in fenestration number in the second term were fit using observed fenestration density at the same time points (6, 10, 15, and 20 weeks) in diabetic mice [10]. Parameters *k*_*s*_ and *k*_*d*_ and time constant ($\tau_{pMLC}$) for pMLC activation were calibrated against observed data for fenestration width in GECs in diabetic mice in [10]. The shape factor (*n*_*f*_) was set at the same value estimated in the first step of the estimation process.

The best-fit parameters were used for model prediction. Parameters relevant to Eqs (4) and (5) are reported in Table 1, and those for the species and reactions in the LBODEs are listed in Tables B and C in S1 Appendix, respectively.

**Table 1:** Parameters that modulate change in fenestration number and diameter.

Parameter	Value	Unit	Source
$k_{form}$	1.01	$hr^{-1}$	estimated
$k_{loss}$	4.61	$hr^{-1}$	estimated
*n*_*f*_	4.00	–	estimated
$y_{Number_{ss1}}$	7.00	–	estimated
$y_{Number_{ss2}}$	4.02	–	estimated
*k*_*s*_	65.9	nm $hr^{-1}$	estimated
*k*_*d*_	2.04	$hr^{-1}$	estimated
$pMLC_{0}$	0	–	–
$y_{Diameter_{0}}$	47.91	nm	[10]
$\tau_{pMLC}$	400.0	hr	estimated

### Uncertainty quantification

We quantified the uncertainty in the model predictions for fenestration diameter and number using a Monte Carlo ensemble simulation, a form of sampling-based uncertainty propagation [27,76,77]. The fitted parameter sets from the multi-start parameter estimation were labeled as an acceptable subset if the SSE for a given parameter subset was within 20% of the lowest SSE for the best-fit parameters. To estimate the parameters’ uncertainty, we used the function randsample in MATLAB to return a user-specified number of values sampled uniformly at random from the values in the vector population. For each parameter, we specified population as the values of that parameter in the acceptable parameters subset. We called randsample independently for each parameter to generate 100 samples. The resulting 100 values for each parameter are considered as the posterior distribution for the parameter. The model in Eqs (3)-(5) and Eqs (S1)-(S34) in S1 Appendix was run using the single mean glucose trajectory *G*(*t*) (Fig 2) as the input for each parameter combination of the 100 samples from the parameter posterior distributions to predict the model outputs of fenestration number and diameter. The posterior distributions of predictions for each output were used to calculate the 95% equal-tail credible interval using quantiles at each time point, which defines the region where there is a 95% probability of containing a true estimate [27,78,79].

### Sensitivity analysis to determine targets for therapeutic interventions

We analyzed the model in Eqs (3)-(5) and Eqs (S1)-(S34) in S1 Appendix under various simulated perturbed activity levels to screen for new and potential strategies for therapeutic intervention. We focused on two modes of therapeutic intervention: (1) protein knockdown and (2) reducing the strength of a reaction. We compared the sensitivity of each species node and reaction in the network to perturbations in $y_{{max}_{i}}$ and *W*_*j*_. Knockdown of species *i* is achieved by reducing $y_{{max}_{i}}$. Decreasing the reaction weight (*W*_*j*_) reduces the strength of the reaction. The local sensitivity index is defined as

$$S_{m,k}=\frac{\Delta Y_{m}}{\Delta P_{k}}\frac{P_{k}}{Y_{m}}$$ (6)

where *S*_*m*,*k*_ is the normalized sensitivity coefficient for a given output *m* and parameter *k*, *Y*_*m*_ is the value of the output *m* at the optimal parameter values *P*, $\Delta P_{k}$ is a perturbation in parameter *k*, and $\Delta Y_{m}=Y_{m}(P\;+\;\Delta P_{k})\;-\;Y_{m}(P)$ is the change in output *m* calculated at the perturbed parameter value.

For the sensitivity analysis results presented, Eq (6) was used to calculate the local sensitivity index for fenestration number and fenestration diameter as the outputs when each species and reaction parameter related to interventions—$y_{{max}_{i}}$ and *W*_*j*_, respectively—in the LBODEs model in Eqs (S1)-(S34) in S1 Appendix was reduced one-at-a-time by 100% from its optimal value (Tables B and C in S1 Appendix), simulating complete knockdown of a species or complete inhibition of a reaction for the entire duration of the simulation, effectively removing that species or reaction from the PSN. Note that these simulations used the single mean glucose trajectory *G*(*t*) (Fig 2) input. We assessed the sensitivity indices to elucidate the functional effects of each node and reaction on fenestration structure.

### *In silico* interventions

We considered three types of *in silico* interventions to “treat” our virtual mouse population:

1. Time-dependent glucose control.
2. Knockout of known targets for ECs using chemical agents.
3. Time-dependent perturbation tests on the PSN.

*In silico* interventions were simulated to identify the functional influence of each node under high glucose conditions and the responses to various glucose trajectories. Similar *in silico* knockdown experiments and sensitivity analyses have been commonly used to study large network models [30,32,33]. Currently, no treatment strategies mitigate the loss of GEC fenestration in diabetic kidneys by leveraging precise mechanisms of action. *In silico* tests could identify disease intervention strategies and mechanisms to regulate endothelial dysfunction.

#### Time-dependent glucose control.

For intervention by time-dependent glucose control, we simulated the model using the best-fit parameters while changing the dynamic glucose stimulus. We used the 100 *G*(*t*) trajectories for inter-subject variability (Fig 3) defined earlier for all cases at times before a glucose “control” intervention was applied. After the time point for glucose control intervention *t*_*c*_, G(t)=min(G(t)) and $W_{GLU}^{\prime }(t)=0$ for $t\geq t_{c}$. We explored the effects of multiple values of *t*_*c*_ at 4 and 10 weeks on downstream species and fenestration structure. We also quantified the change in each species and the fenestration number and diameter relative to the baseline as the difference in values between 20 weeks and the initial time. Each of the values was taken as the mean simulated output of the virtual mouse population (*n* = 100) using glucose inter-subject variability in the input.

#### Knockout of known targets for endothelial cells using chemical agents.

A recent study demonstrated the dose-dependent role of several chemical agents in regulating fenestration porosity and diameter of LSECs [26]. As a second type of *in silico* intervention, we simulated the effects of the chemical agents on the GEC fenestration structure using the extended LBODEs model. Five chemical agents KN93, ML-7, Y27632, calyculin A, and cytochalasin B [26] have known inhibitory actions on targets calcium, MLCK, Rho/Rock, MLCP, and stressed actin fibers, respectively. We have summarized the experimentally observed effects of these chemical agents in LSECs in Table D in S1 Appendix. We simulated the effects of these chemical agents by *in silico* species inhibition by reducing $y_{{max}_{i}}$ for the respective target species to 0 at the initial time. Glucose inter-subject variability was considered. A statistical pairwise Student’s t-test [80] was used to compare the differences in means of fenestration number and diameter at 20 weeks between healthy and diabetic mice [10] and simulated diseased groups with or without treatment using ttest2 in MATLAB, which uses observed or model-predicted values in each group to compute a mean, variance, and sample size for each group and compare them. Therefore, the extended LBODEs model was simulated for each treatment’s effects on the virtual mouse population (*n* = 100) using the *G*(*t*) trajectories for inter-subject variability (Fig 3). For each pairwise t-test, a significant difference between groups was reported when the calculated p-value is below the 0.05 significance level. We also considered that the inhibition from the chemical agents could be partial and applied at different times. We did not do statistical tests on these comparisons, but we do provide dynamic plots of the results.

#### Time-dependent perturbation tests on the PSN.

The third type of *in silico* intervention we considered was a more exploratory time-dependent series of perturbation tests on the intervention-related parameters of the PSN. These simulations used the single mean glucose trajectory *G*(*t*) (Fig 2) input. Similar to the sensitivity analysis, the fenestration number and fenestration diameter were predicted when species and reaction parameters—$y_{{max}_{i}}$ and *W*_*j*_, respectively—in the LBODEs model in Eqs (S1)-(S34) in S1 Appendix were perturbed by a one-at-a-time reduction from the optimal values (Tables B and C in S1 Appendix). However, here each inhibition was only by a 50% reduction in the respective parameter for a partial knockdown, and the interventions were applied at 8, 10, or 20 weeks rather than at the beginning of the simulation as in the sensitivity analysis. To determine the effects after interventions at 20 weeks, all these interventions were simulated until 30 weeks (5040 hours). We kept the glucose stimulus constant at the final data point for the interval after 20 weeks, i.e., G(t)=G(20weeks) for t≥20 weeks. We grouped the results by sensitive and non-sensitive species and reactions for visualizations.

## Results

### Model simulations of disease onset and progression

**Fig 4:**
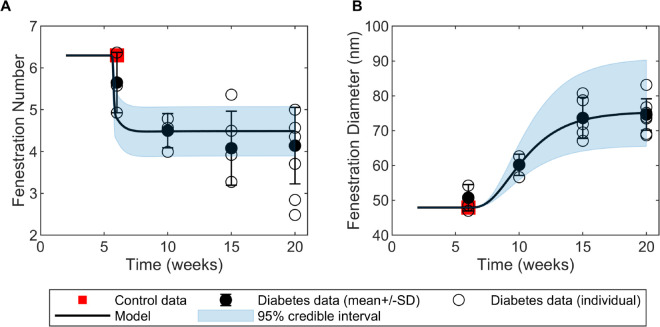
Simulated fenestration number and diameter. A: Simulated fenestration number fitted against observed mean fenestration density (black circles) in diabetic mice [10]. B: Simulated fenestration diameter fitted against observed mean fenestration width (black circles) in diabetic mice [10]. Blue-shaded regions show the 95% credible intervals of the predictions. Data are shown as mean ± standard deviation (SD). Individual fenestration width and density are also reported for each diabetic mouse (open circles). Initial values for fenestration diameter and number were assumed to be the same as baseline control mean data values of fenestration width and density (red squares) for healthy mice in Finch et al. [10].

Using the glucose dynamics shown in Fig 2 for the single mean glucose trajectory *G*(*t*) with simulated glucose levels in the piecewise constant function at the observed means of the data in diabetic mice [10], we calibrated the predictions for the fenestration number Eq (4) and the fenestration diameter Eq (5) to measured data from Finch et al. [10] and quantified the uncertainty in the form of credible intervals (Fig 4). The full model dynamic outputs for each species activity in response to the dynamic glucose stimulus are available in Fig B in S1 Appendix. Fenestration number (Fig 4A) decreased as early as 6 weeks, which coincided with full activation ($W_{GLU}^{\prime }\geq 1$) of normalized glucose around 6 weeks (Fig 2) and subsequent signal transduction and protein activation in the network (Fig B in S1 Appendix). Similarly, we observed an increase in the fenestration diameter in agreement with the experimental data (Fig 4B). The credible intervals are reasonable given the variance of the data (Fig 4).

### Glucose variability effects on fenestration number and diameter

**Fig 5:**
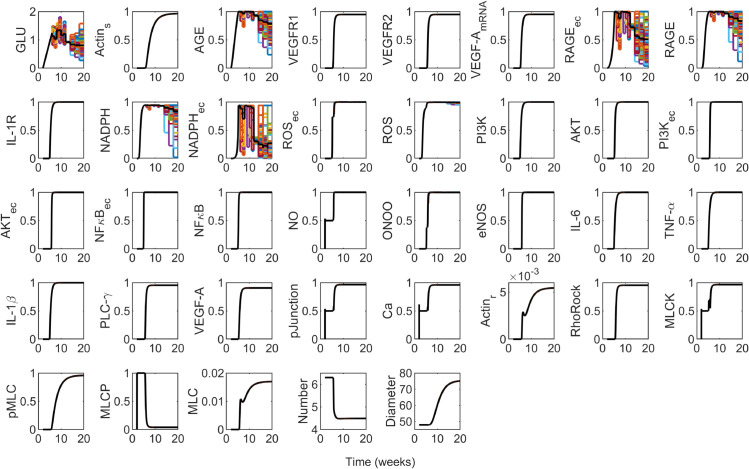
Predicted dynamics of the species in the multi-cellular protein interaction network (Fig 1) simulated using the 100 G(t) trajectories (Fig 3) for the virtual mouse population as input. Note that the means of the outputs from the 100 input glucose trajectories are shown in black, while the dynamic outputs for individuals cycle through MATLAB’s default color order.

Next, we explored the effects of inter-subject variability in glucose concentration input using the 100 *G*(*t*) trajectories (Fig 3 and the first panel of Fig 5) for the virtual mouse population. We simulated the effects on the changes in species activity and fenestration structure. As an alternative visualization of the same results plotted in Fig 5, we created histograms for the distributions of the glucose activity GLU and the structural response variables (fenestration number and diameter) for the virtual mouse population in each time interval for 6–20 weeks (Fig C in S1 Appendix). Note that the MATLAB ordinary differential equation solver (ode15s)’s default tolerance of 10^−6^ was used, so distributions that vary within the range of a value only at the 6th significant figure are essentially the same within the tolerance of the technique for solving the model equations. This is characteristic of the distributions for fenestration number for all times in 6–20 weeks (second column of Fig C in S1 Appendix) and for the fenestration diameter at week 7 (third column, second row of Fig C in S1 Appendix).

We observed no substantial variations in fenestration dynamics and most species due to glucose inter-subject variability (Fig 5). The activities for the nodes AGE, RAGE_ec_, RAGE, NADPH, and NADPH_ec_ varied extensively with glucose variations (Fig 5). The variability in glucose concentration was mainly observed in the hyperglycemic range, indicating impaired glucose tolerance.

### Sensitive targets for interventions

**Fig 6:**
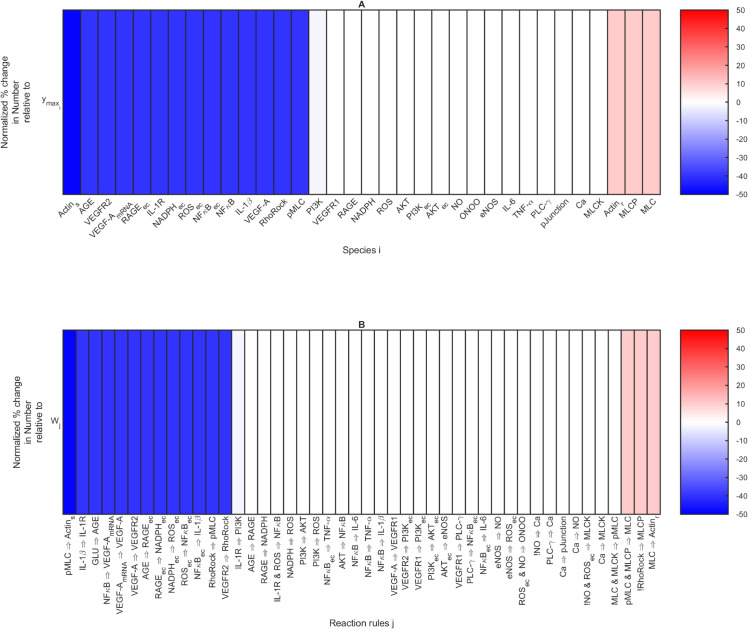
Ordered normalized sensitivity indices in Eq (6) with respect to fenestration number output expressed as percentages. A: Sensitivity indices $S_{y_{Number},y_{{max}_{i}}}$ for species *i* when $y_{{max}_{i}}$ was completely inhibited at the initial time. B: Sensitivity indices $S_{y_{Number},W_{j}}$ for reaction *j* when *W*_*j*_ was completely inhibited at the initial time.

**Fig 7:**
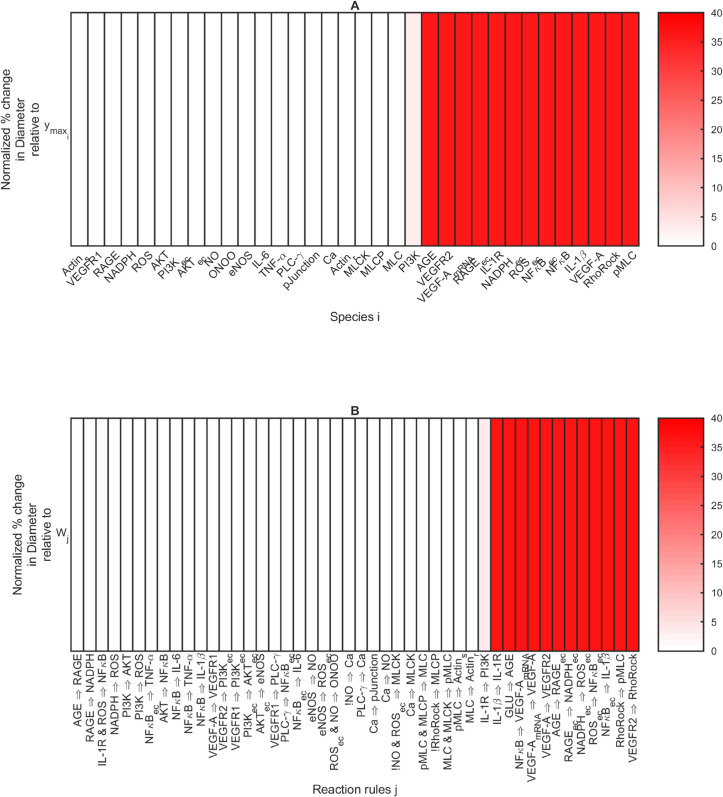
Ordered normalized sensitivity indices in Eq (6) with respect to fenestration diameter output expressed as percentages. A: Sensitivity indices $S_{y_{Diameter},y_{{max}_{i}}}$ for species *i* when $y_{{max}_{i}}$ was completely inhibited at the initial time. B: Sensitivity indices $S_{y_{Diameter},W_{j}}$ for reaction *j* when *W*_*j*_ was completely inhibited at the initial time.

We created heatmaps to summarize the sensitivity analysis results (Figs 6 and 7). In each panel, the bars are colored by the normalized sensitivity index from Eq (6) expressed as percentages. The values are sorted from low to high. We considered all normalized sensitivity indices $S_{m,k}>1.5\%$ to be “sensitive,” and values below the threshold to be “non-sensitive.” We generated four sensitive sets:

1. Species *i* that are sensitive with respect to the fenestration number output when $y_{{max}_{i}}$ is completely inhibited: $S_{y_{Number},y_{{max}_{i}}}>1.5\%$ (Fig 6A).
2. Reaction *j* that are sensitive with respect to the fenestration number output when *W*_*j*_ is completely inhibited: $S_{y_{Number},W_{j}}>1.5\%$ (Fig 6B).
3. Species *i* that are sensitive with respect to the fenestration diameter output when $y_{{max}_{i}}$ is completely inhibited: $S_{y_{Diameter},y_{{max}_{i}}}>1.5\%$ (Fig 7A).
4. Reaction *j* that are sensitive with respect to the fenestration diameter output when *W*_*j*_ is completely inhibited: $S_{y_{Diameter},W_{j}}>1.5\%$ (Fig 7B).

The sensitive set with respect to the fenestration number output from Fig 6A includes species Actin_s_ through PI3K on the blue side and Actin_r_ through MCL on the red side. The PI3K value is -2 and appears as a faint blue color. The sensitive set from Fig 6B includes the 13 reactions with vibrant blue bars, the three reactions with pink bars, and IL-1R ⇒ PI3K (*j* = 7), which has a faint blue bar and a value of -2 %. The cutoff for the sensitive sets was determined by observation of Fig D in S1 Appendix. PI3K and IL-1R ⇒ PI3K inhibition have noticeable effects on the corresponding curves of Figs D.A and D.B in S1 Appendix. On the contrary, none of the non-sensitive results were substantially different than the no inhibition cases (Fig E in S1 Appendix).

Similarly for the fenestration diameter output, PI3K and IL-1R ⇒ PI3K were on the cutoff of inclusion vs. exclusion from the sensitive sets based on Fig 7, where they have values of 3 % and appear in light shades of pink. The sensitive set from Fig 7A includes species PI3K through pMLC. The sensitive set from Fig 7B includes IL-1R ⇒ PI3K and the 12 reactions with vibrant red bars. PI3K and IL-1R ⇒ PI3K inhibition have noticeable effects on the corresponding curves of Figs D.C and D.D in S1 Appendix.

The magnitude of the sensitivity index indicates the degree of change in output (fenestration number or diameter) at the final time relative to the change in parameter at the initial time. Because we only considered decreases in parameters, a positive sign of the sensitivity index means that the model predicted a smaller value of the output than the no inhibition case with a decrease in parameter value, and a negative sign indicates a larger value of the output compared to the no inhibition case with a decrease in the parameter value.

For the fenestration number, MLCP, MLC, and relaxed actin fibers had positive values of the sensitivity index (Fig 6A), and their inhibition decreased the fenestration number further than the no inhibition case (Fig D.A in S1 Appendix). The remaining sensitive species for fenestration number increased the fenestration number relative to the no inhibition case, i.e., they had negative values of the sensitivity index (Fig 6A). Noticeably, inhibition of stressed actin fibers Actin_s_ led to an increase in fenestration number beyond the healthy initial level (Fig D.A in S1 Appendix). Complete inhibition of AGE, VEGFR2, VEGF-A (mRNA), RAGE_ec_, IL-1R, NADPH_ec_, ROS_ec_, RAGE_ec_, NF_*κ*_B_ec_, NF_*κ*_B, IL-1β, VEGF-A, Rho/Rock, and pMLC allowed for the fenestration number to remain at the initial condition (healthy case), thus no disease condition was developed for these sensitive species. The fenestration number dynamics were similar for the sensitive reactions (Fig D.B in S1 Appendix) as for the sensitive species (Fig D.A in S1 Appendix) because the sensitive reactions for the fenestration number are those that activate the sensitive species (Fig 6B).

The sensitivity analysis with respect to the fenestration diameter (Fig 7) yielded sensitive parameters with all positive sensitivity index values. PI3K and IL-1R ⇒ PI3K were the only sensitive species or reactions that did not maintain the diameter at the healthy initial condition after their inhibition. We found that the following species were most effective in controlling fenestration diameter when controlled early in diabetic mice (Figs 7 and D.C in S1 Appendix): AGE, VEGFR2, VEGF-A (mRNA), RAGE_ec_, IL-1R, NADPH_ec_, ROS_ec_, RAGE_ec_, NFκB_ec_, NFκB, IL-1β, VEGF-A, RhoRock, and pMLC. The following reactions maintained fenestration diameter at its healthy initial value (Figs 7 and D.D in S1 Appendix): IL-1β ⇒ IL-1R, GLU ⇒ AGE, NFκB ⇒ VEGF-A (mRNA), VEGF-A (mRNA) ⇒ VEGF-A, VEGF-A ⇒ VEGFR2, AGE ⇒ RAGE_ec_, RAGE_ec_ ⇒ NADPH_ec_, NADPH_ec_ ⇒ ROS_ec_, ROS_ec_ ⇒ NFκB_ec_, NFκB_ec_ ⇒ IL-1β, RhoRock ⇒ pMLC, and VEGFR2 ⇒ RhoRock.

### Proposed treatment and intervention strategies

The extended LBODEs model was helpful in making predictions about potential disease interventions and treatments. In the following, we show the results from the three types of *in silico* interventions with the details defined in the Methods.

#### Glucose control starting at different times.

**Fig 8:**
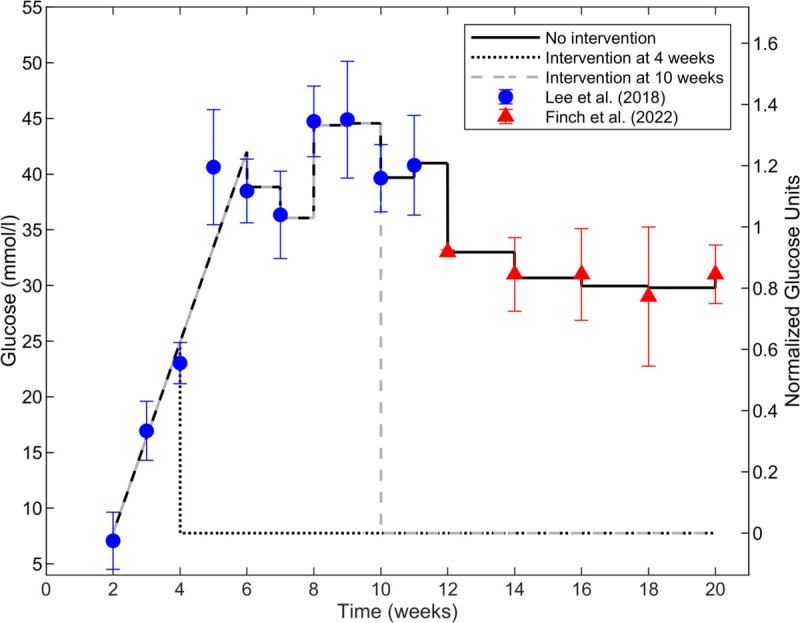
Simulated glucose input concentration profiles for time-dependent glucose control interventions. For 6–20 weeks, the 100 *G*(*t*) trajectories (Fig 3) for the virtual mouse population were used as the inputs before and without intervention, and the means of the 100 profiles are plotted. The “No intervention” case (solid black line) is the glucose inter-subject variability scenario. *In silico* glucose control “intervention" was applied at 4 weeks (dotted black line) and 10 weeks (dashed gray line) by resetting glucose to its initial value G(t)=min(G(t)). Glucose concentration data values are from Lee et al. [22] (blue circles) and Finch et al. [10] (red triangles). Data are shown as means ± standard deviations.

In mice carrying the diabetes mutation (leptin-deficient), the manifestation of the diabetic syndrome depends on genetic background. In these mice, used as the reference for this study, male mice developed diabetes around 6 weeks of age [81–83]. In Fig 8, glucose levels were controlled *in silico* at time points before (*t* = 4 weeks) and after (*t* = 10 weeks) the mice reached the diabetic state.

**Fig 9:**
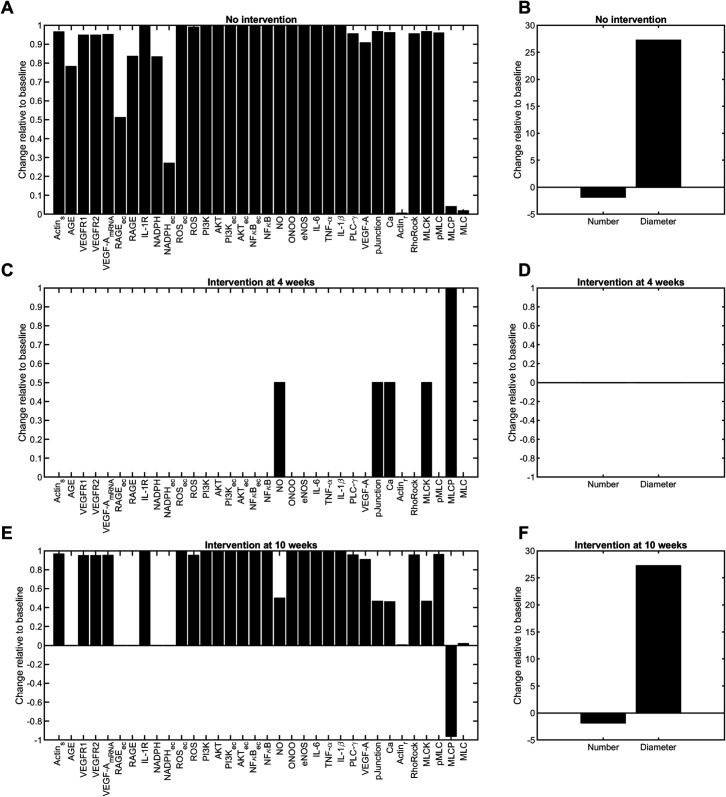
Predicted changes in species in the multi-cellular protein interaction network (Fig 1) simulated using the time-dependent glucose control interventions (Fig 8) as input. Predicted changes in A: activity and B: fenestration number and diameter from baseline without glucose intervention. Predicted changes in C: activity and D: fenestration number and diameter from baseline with glucose intervention applied at 4 weeks. Predicted changes in E: activity and F: fenestration number and diameter from baseline with glucose intervention applied at 10 weeks.

Fig 9 shows the changes in species activity and structural outputs relative to baseline (initial conditions) in response to the various glucose interventions (Fig 8B). The full model dynamic outputs for each species activity in response to the various glucose stimuli (no intervention and glucose control interventions at 4 and 8 weeks) are available. For the no intervention case for the single mean glucose trajectory *G*(*t*) (Fig 2) as the input, the dynamic outputs are in Fig B in S1 Appendix. For the no intervention case for the inter-subject variability (which begins at 6 weeks) using the 100 *G*(*t*) (Fig 3) input trajectories for the virtual mouse population, the dynamic outputs are in Fig 5. These results are summarized in Fig 9A and 9B. For glucose control intervention at 4 weeks (Fig 8), the dynamic outputs are in Fig F in S1 Appendix. The results from each of the panels are summarized in Fig 9C and 9D. For glucose control intervention at 10 weeks with inter-subject variability (Fig 8), the dynamic outputs are in Fig G in S1 Appendix. These results are summarized in Fig 9E and 9F.

The model predicted that glucose intervention at 4 weeks prevented upregulated protein expression and changes in fenestration structure (Fig 9C and 9D). Glucose control at 4 weeks maintained balance in NO and calcium activity (Fig F in S1 Appendix), which may be relevant in regulating downstream signaling dysfunction. Comparing no intervention and intervention at 4 weeks changes (Fig 9A and 9C), MLCK protein levels decreased, and MLCP protein levels increased considerably upon glucose intervention. On the contrary, glucose intervention at 10 weeks was ineffective in controlling fenestration dynamics (Fig 9F); however, it suppressed the upregulation of AGE, RAGE, and NADPH activity in macrophages and GECs (Fig 9E). The normalized glucose levels reached their maximal activity ($W_{GLU}^{\prime }$ = 1) by 5 weeks (Fig 8 and the first panel in Fig G in S1 Appendix), which led to a self-sustained maximal activation of the species in the network (Fig G in S1 Appendix). The activity of most species in the network at 5 weeks (Fig G in S1 Appendix) was equal to or above their *EC*_50_ (Table C in S1 Appendix). The positive feedback loops in the network also regulated the self-sustained maximum activity of downstream species. This resulted in consistently dysregulated signaling, loss of fenestrations, and an increase in fenestration diameter. Together, these results suggest that glucose control may be an effective strategy for controlling and mediating GEC activation in the early stages of DKD. Yet, it cannot be used as the only strategy to modulate complex signals and pathways that regulate fenestrations in the later stages of DKD.

#### Knockout of known endothelial cell targets with chemical agents.

**Fig 10:**
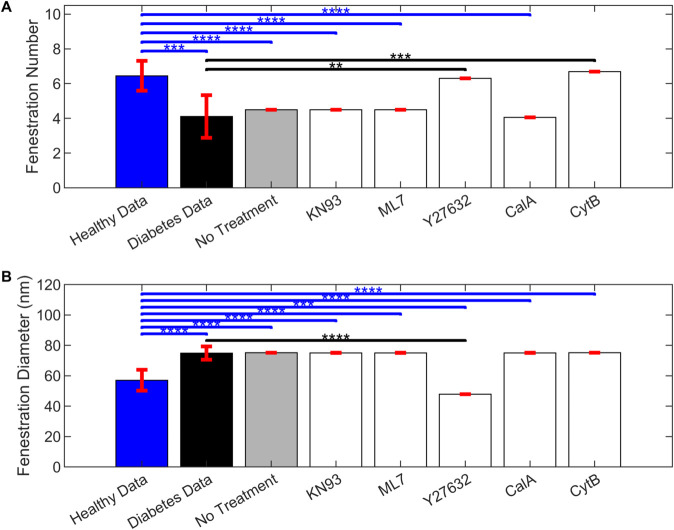
Bar plots comparing the simulated glomerular endothelial cell fenestration number and diameter. A: fenestration number and B: diameter at 20 weeks in the inter-subject glucose variability virtual mouse population without (gray bars) and with treatment by respective chemical agents (white bars). For the *in silico* treatment, each of the targeted species parameters ($y_{max_{i}}$) was reduced one-at-a-time by 100% at the initial time of 2 weeks. Observed number and diameter in healthy (blue bars) and diabetic (black bars) mice from Finch et al. [10] are also shown as mean ± standard deviation of the data. ***: p-value<0.001, ****: p-value<0.0001 for t-test comparison between data for healthy mice and model predictions in diseased virtual mice with or without treatment. **: p-value<0.01, ***: p-value<0.001, ****: p-value<0.0001 for t-test comparison between data for diabetic mice and model predictions in diseased virtual mice with or without treatment. Simulated results bars are shown as mean ± standard deviation across the virtual mouse population (*n* = 100).

We compared the effects of KN93, ML-7, Y27632, calyculin A (CalA), and cytochalasin B (CytB) on their respective targets—calcium, MLCK, Rho/Rock, MLCP, and stressed actin fibers, respectively—that we assumed were shared between fenestrated endothelial cell types LSECs and GECs (Table D in S1 Appendix). Fig 10 compares the simulated GEC fenestration diameter and number in the virtual mouse population at 20 weeks without treatment, with *in silico* treatment with these chemical agents at the start of the simulation (2 weeks), and reference values observed in healthy and diabetic mice. We used the virtual mouse population for our *in silico* knockout tests. The glucose input distributions are in Figs 3 and C in S1 Appendix. The means and standard deviation values of fenestration number and diameter for each simulated condition are available in Table E in S1 Appendix for the virtual mouse population.

Fenestration number significantly increased upon treatment with Y27632 and cytochalasin B (via inhibiting Rho/Rock and Actin_s_) compared to diabetic mice and restored the fenestration number back to the healthy level (Fig 10A). When MLCP protein was inhibited by calyculin A, the predicted fenestration number decreased further than the no treatment case, but the difference between the calyculin A treatment and the diabetic mice data was not statistically significant (Fig 10A). No significant changes were observed in the fenestration number upon treatment with KN93 and ML-7 compared to the no treatment case (Fig 10A). Diabetic mice, the no treatment case, and treatments with KN93, ML-7, and CalA were significantly different than the healthy control data, but not different from the diabetic mice data (Fig 10A).

The fenestration diameter decreased significantly upon inhibition of Rho/Rock using Y27632, and the predicted value was significantly lower than the mean for healthy mice (Fig 10B). The other treatments KN93, ML-7, calyculin A, and cytochalasin B were ineffective in regulating the GEC fenestration diameter and were not statistically different than the diabetic mice or the no treatment case (Fig 10B).

When comparing the knockout results in Fig 10 with the sensitivity analysis results in Figs 6 and 7, the results were clearly explained. Y27632 targets Rho/Rock, which was sensitive for both fenestration number and diameter (Figs 6 and 7). Complete inhibition of Rho/Rock returned the fenestration number (Fig D.A in S1 Appendix) and fenestration diameter (Fig D.C in S1 Appendix) to their baseline values. Cytochalasin B targets Actin_s_, which had the largest negative sensitivity index for fenestration number (Fig 6) and went beyond returning the number to the baseline (Fig D.A in S1 Appendix). Calyculin A targets MCLP, which had a positive sensitivity index for fenestration number (Fig 6) and lowered the fenestration number further than the no inhibition case (Fig D.A in S1 Appendix). KN93 and ML-7 target Ca and MLCK, respectively, and were not among the sensitive species for fenestration number or diameter (Figs 6,7, E.A, and E.C in S1 Appendix). Additionally, Actin_s_ and MCLP were not sensitive species for fenestration diameter (Fig E.C in S1 Appendix).

#### Exploratory perturbation of all possible targets in the network starting at different times.

In most studies, it was reported that diabetes may develop and progress into diabetic kidney disease around 10–12 weeks in diabetic mice [22]. Therefore, it may be relevant to understand when to intervene as DKD develops and progresses in these subjects and to effectively regulate fenestration number and diameter before damage worsens. To study this *in silico*, we inhibited by 50% the influential species and reactions identified by the sensitivity analysis at 8, 10, and 20 weeks to compare the effects on fenestration structure during pre-DKD, early-DKD, and late-DKD stages in diabetic mice, respectively. The dynamic effects of inhibiting these species and interactions on fenestration structure during the early and late stages of DKD in mice were simulated.

We considered the effects of the perturbations to the sensitive targets for the chemical agents discussed in the previous section when applied at different times. Fig H in S1 Appendix combines the sensitive analysis results with full inhibition at 2 weeks and the dynamic perturbation analysis results with 50% inhibition at 8, 12, or 20 weeks. As a reminder, cytochalasin B targets Actin_s_, calyculin A targets MCLP, and Y27632 targets Rho/Rock. Notably, partial inhibition was insufficient for returning fenestration number or diameter to their baseline values for any of the sensitive targets (Fig H in S1 Appendix). However, Rho/Rock partial inhibition did consistently lower the fenestration diameter to just 11 % above the baseline value by 30 weeks for any of the tested intervention times (Fig H.B in S1 Appendix).

**Fig 11:**
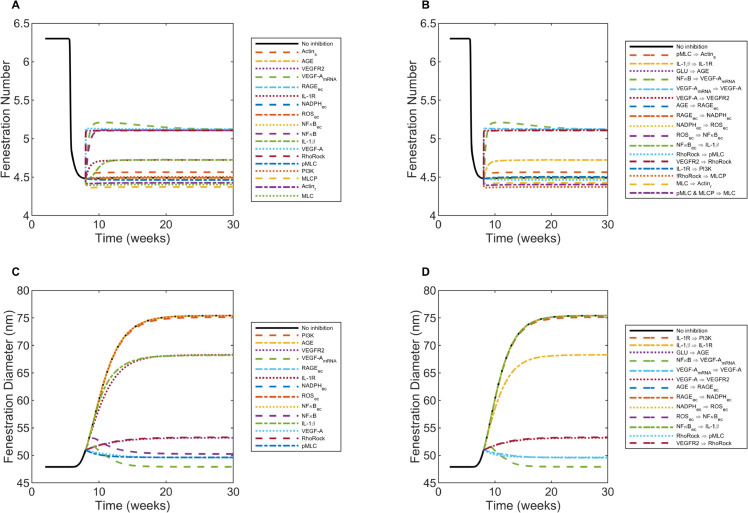
Structural effects over time as a result of the perturbation analysis for inhibiting sensitive parameters at 8 weeks. After starting at their optimal values, each of the species parameters ($y_{max_{i}}$) and reaction parameters (*W*_*j*_) was reduced one-at-a-time by 50%. A, B: Fenestration number output for perturbed parameters for sensitive A: species and B: reactions. C, D: Fenestration diameter output for perturbed parameters for sensitive C: species and D: reactions. Black curves (labeled as “No inhibition”) on each panel serve as the controls and show the structural effects without inhibition.

For an *in silico* 50% knockdown of all sensitive species at 8 weeks in diabetic mice, a noticeable increase in fenestration number was achieved with 50% inhibition of Actin_s_, VEGFR2, VEGF-A (mRNA), IL-1R, NFκB, IL-1β, VEGF-A, and RhoRock (Fig 11A). A modest, immediate decrease in fenestration number was achieved by inhibiting the activity of MLC, MLCP, and Actin_r_ (Fig 11A). NADPH_ec_ inhibition gradually led to a modest decrease in fenestration number by 30 weeks (Fig 11A). The other sensitive species did not deviate from the fenestration number dynamics for the no inhibition case (Fig 11A), which was also true for the insensitive species (Fig I.A in S1 Appendix).

Upon reducing the strength of interactions for reactions VEGFR2 ⇒ RhoRock, VEGF-A_mRNA_ ⇒ VEGF-A, VEGF-A ⇒ VEGFR2, NFκB ⇒ VEGF-A_mRNA_, and IL-1β ⇒ IL-1R, substantial increases in fenestration number were observed (Fig 11B). The other sensitive reactions modulated the fenestration number within ± 2.7% of the no inhibition case (Fig 11B) and were not therapeutically relevant. The non-sensitive reactions did not deviate from fenestration number dynamics for the no inhibition case (Fig I.B in S1 Appendix).

The 50% inhibition of RhoRock, VEGF-A, VEGFR2, pMLC, VEGF-A (mRNA), and NFκB restored fenestration diameter effectively, while the effects of IL-1β and IL-1R on reducing fenestration diameter were more modest (Fig 11C). The predicted fenestration diameter dynamics for the other sensitive species and the non-sensitive species did not differ appreciably from the no inhibition case (Figs 11C and I.C in S1 Appendix).

Reducing the strengths of interactions for reactions NFκB ⇒ VEGF-A_mRNA_, RhoRock ⇒ pMLC, VEGF-A_mRNA_ ⇒ VEGF-A, VEGF-A ⇒ VEGFR2, and VEGFR2 ⇒ RhoRock were most effective in restoring fenestration diameter (Fig 11D). IL-1β ⇒ IL-1R had a more modest effect on lowering fenestration diameter (Fig 11D). The predicted fenestration diameter dynamics for the other sensitive reactions and the non-sensitive reactions did not differ appreciably from the no inhibition case (Figs 11D and I.D in S1 Appendix).

The inhibition time (8, 12, or 20 weeks) did not significantly affect the final value at 30 weeks for the fenestration number or diameter (Figs 11, H, and I-M in S1 Appendix). In comparison to the cases with partial inhibition at 8 weeks just described in detail (Figs 11 and I in S1 Appendix), we observed similar effects of inhibited species and reduced reaction strengths on fenestration number and diameter for sensitive sets (Fig J in S1 Appendix) and non-sensitive sets (Fig K in S1 Appendix) inhibited partially at 10 weeks and for sensitive sets (Fig L in S1 Appendix) and non-sensitive sets (Fig M in S1 Appendix) inhibited partially at 20 weeks.

## Discussion

Glomerular endothelial activation and dysfunction are early signs of DKD development and progression. Alterations in the size and density of GEC fenestrations have been recently associated with the disruption in glomerular filtration and the progression of diabetic kidney disease [10]. Currently, significant barriers impede understanding the cellular mechanisms that regulate GEC fenestrations, which include a lack of ideal *in vitro* models, fenestration loss in culture, and inconsistencies between *in vitro* and *in vivo* findings [9]. The small size of endothelial cell fenestrations is beyond the limit of resolution of light microscopy and needs newer and advanced technology to be studied accurately. Hence, there is a lack of high-quality quantitative data on transient changes in fenestration size and density in diseased states. It is beneficial to leverage mathematical modeling to study GEC fenestrations and explore signaling drivers that affect their size and density. In this study, we presented an extension to our previously developed protein-protein signaling network model for cross talk between GECs and macrophages. Here, we studied the effects of glucose-mediated signaling dysregulation on structural changes in fenestrated GECs using an *in silico* approach.

Signaling drivers and mechanisms were derived from fenestrated LSECs and considered in the presented model. The interplays between pathways modulating MLCK, Rho/Rock, calcium, NO/eNOS, VEGF/VEGFR, ROS, and actin structure were crucial in understanding fenestration dynamics. Under a changing extracellular environment, actin structures are disassembled and remodeled to balance the structural and functional integrity of the fenestrations [14]. Stresses within the actin structure are correlated with GECs’ fenestration density, and MLC protein phosphorylation is associated with the enlargement of fenestrations [26]. We demonstrated that glucose intolerance and hyperglycemia in diabetic mice resulted in the loss of approximately half of the fenestrations and a 70% increase in fenestration size in GECs from baseline. Previous studies also reported changes in fenestration in the glomeruli in patients diagnosed with diabetic nephropathy [10]. Fenestration loss and alterations in fenestration width for diabetic nephropathy patients [10] were also quantitatively similar to the predicted changes in diabetic mice.

We showed that gradual increases in glucose levels associated with the development of diabetic conditions were correlated with dysregulated species and changes in the size and density of fenestrations. We observed that inter-subject variability in glucose concentrations in diabetic mice had minimal or no effect on normalized protein activity in macrophages or GECs. Note that these glucose variations were predominantly in the range above normalized activity of 0.5 (Fig 3).

We mathematically related glucose-mediated signaling dysregulation and autocrine inflammatory feedback to loss of fenestration number and increased diameter. Previously published logic-gated network models [32,70] have drawn mathematical relations between protein signaling and normalized changes in the cell area using LBODEs. Compared to previous models, our model related normalized signaling activity to actual changes in fenestration structure in diabetic mice.

We observed that glycemic control in the early stage was most effective in reducing upregulated species and modulating fenestration loss. After glucose concentration reached 25 mmol/l, often considered a high glucose level in mice, glucose control was ineffective for controlling GEC activation, signaling dysregulation, and structure in the later stages of DKD development. We posit that disease intervention strategies beyond glucose control are essential for regulating the complex downstream signals and pathways that govern fenestrations in diseased GECs. This was seen through the predicted imbalanced activity of proteins in the MLC phosphorylation cycle (pMLC, MLCP, MLCK, and MLC) after glucose control (Fig 9).

Using sensitivity analyses, selective *in silico* knockdown of the species provided alternative strategies for disease intervention. Strategies to inhibit stressed actin fibers, VEGF-A (mRNA), VEGF-A, VEGFR2, IL-1R, IL-1β, Rho/Rock, and NFκB were predicted to effectively recover the loss in fenestrations in diseased GECs. Rho/Rock, VEGF-A, VEGFR2, pMLC, and NFκB protein inhibition were expected to be promising strategies for controlling fenestration diameter in diseased GECs.

The presented network model was based on prior evidence on proteins or pathways that regulate fenestrated endothelial structure integrity. Although limited *in vivo* studies have investigated the role of these proteins or pathways on GEC fenestrations, some *in vitro* experiments have demonstrated that treatment of whole glomeruli with cytochalasin B reduced deformation and loss of foot processes in podocytes [84] and increased GEC porosity and pore size [2,85]. Rock protein inhibitors and cytochalasin B noticeably increased porosity in healthy LSECs in a few minutes to hours [16].

Non-selective Rock inhibitors, Fasudil [86] and Y27632, were shown to suppress renal injury and promote renoprotective effects in diabetic mice. The proposed model demonstrates a qualitatively similar regulatory effect of Y27632 on Rock inhibition and cytochalasin B on GEC porosity and pore size(Fig 10). Inhibition of the Rho/Rock pathway also reduced adhesion molecule expression and macrophage infiltration to GECs induced by AGEs in diabetic mice [86]. Y27632 also affected fatty acid utilization and redox balance in mesangial cells in the glomerulus [87]. We conclude that a plausible correlation between the proposed target proteins in this work and glomerular dysfunction, cytoskeletal arrangement, immune cell infiltration, and renal injury exists. The combined effects of Rock inhibitors and depolymerization agents have the potential to be investigated as a treatment strategy in diseased GECs.

No published studies have tested the impacts of targeted inhibition of MLCK, Rock, and MLCP proteins in GECs affected by diabetes. A balance in MLCP and MLCK protein levels may be crucial in regulating fenestration diameter and number (Fig 9). A relatively higher MLCK protein than MLCP protein was observed during fenestration structure damage despite glucose intervention at 10 weeks (Fig 9). A balanced MLCP and MLCK expression should enhance fenestration formation (Fig 6). At the same time, MLCP and MLCK perturbations had potentially no or minimal change in fenestration diameter as seen from the sensitivity analysis (Fig 7). The interdependency of the MLCP and MLCK protein balance is essential due to the positive feedback loops in the MLC phosphorylation cycle. Both MLCK and MLCP proteins regulated the fenestration diameter and porosity in LSECs [26]. Counterintuitive to the expectations, inhibition of MLCK and MLCP by chemical agents like ML-7 and calcyculin A, respectively, resulted in fenestration loss and an increase in fenestration diameter (Table D in S1 Appendix).

*In silico* treatment with KN93 did not affect fenestration diameter and number (Fig 10), but treatment with KN93 has other renoprotective regulatory effects in mice. Targeted delivery of KN93 inhibited Ca2+/calmodulin–dependent kinase 4 (CaMK4) and reduced LPS-induced podocyte injury, mesangial cell proliferation, and proteinuria in mice [88]. Inhibiting CaMK4 using KN93 reduced glycolysis in regulatory T cells through metabolic rewiring and alleviated immune response-mediated renal injury [88–90]. KN93 treatment also reduced adhesive and migratory function in neutrophils and diminished CD4+ T cell population associated with renal inflammation [91,92].

We acknowledge the limitations of the model and the potential for further improvement. The protein activity is quantified as a fractional activation or inhibition rather than absolute quantities, such as concentration, and is limited to analyses with normalized chemical species levels. However, the predicted normalized activity of a species can be transformed into its actual values if needed. Alternative mathematical functions could be used to define the protein-protein interactions instead of the normalized Hill-type function to capture other cellular and molecular dynamics. Additionally, activity was used as a way to lump both intensity from single cells and from variable numbers of cells. These could be decoupled by explicit considerations of the dynamics of the numbers of infiltrating macrophages.

We considered a range of weekly or biweekly glucose fluctuations in the model, with limited effects on the output. It would be possible to simulate glucose profiles on an hourly timescale that represent a broad population of diabetic patients; however, additional model considerations, such as meal intake, glucose distribution, and glucose clearance, would be required to simulate accurate glucose profiles in a diabetic population. Future improvements, including availability and integration of more cell-specific clinical data, would certainly enhance the capabilities of the present model to represent diabetic patient populations.

In this model, the fenestration number was considered as a continuum, representing an average value across all regions, not a discrete integer number of fenestrations. VEGF-A is the only mediator of macrophage-GEC communication and GEC activation in the network model, a major assumption and limitation of the model, which is based on prior evidence on the role of VEGF and its receptors in maintaining GEC structure and functional integrity [28]. Although podocytes in the kidney mainly regulate VEGF-mediated GEC activation [93], in the absence of podocytes, we considered macrophages to be the main source of VEGF-A and VEGF-mediated GEC activation. Additionally, autocrine feedback between cytokines (IFN-γ, IL-1β, and TNF-α) and their receptors could also activate GECs and initiate communication between macrophages and GECs; however, this is not considered in the model [94,95]. The diseased model predictions are limited to the pro-inflammatory phenotype (M1-like) of macrophages and do not represent the individual cell-level interactions between macrophages and the GEC population. The calibration and validation of the logic-based modeling framework were limited to *in vitro* and *in vivo* mouse studies.

Moreover, among emerging or existing therapies being tested to control DKD progression, the SGLT2 inhibitor empagliflozin restored GEC fenestration density in leptin-deficient mice despite no expression of SGLT2 in GECs in these mice [19]. Although no clear mechanisms linking SGLT2 inhibition and fenestration structure are known yet, changes in expressions of PV-1, Caveolin-1, and EHD3 were implicated in regulating permeability through GECs [9,10,19]. These proteins are implicated in diaphragm formation in GEC fenestrations. Our research assumes that mature GECs do not possess diaphragms [5]. However, if sufficient experimental evidence emerges, it could be useful to investigate the mechanistic role of the PV-1, Caveolin-1, and/or EHD3 on fenestration structure and function in GECs.

There is a lack of knowledge related to the fundamental biology of the regulating GECs. Advanced imaging techniques known as super-resolution microscopy may offer the potential to facilitate the accurate measurement of fenestrations in GECs [9]. Technological advancements in 3-dimensional (3D) glomerular organ-on-a-chip to precisely study cell–cell or cell–matrix and soluble mediators within the glomerular microenvironment under physiologically relevant flow rates and shear stresses [96] could also expand the translatability of glomerular biology under pathophysiologic conditions. The model accuracy can be further improved through validation with other *in vitro* or *in vivo* data as available or through emerging data from single-cell analyses. Further, capturing individual cell-level dynamics and molecular signaling within each cell may be useful in understanding the macrophage phenotypic landscape over time [97] as the disease progresses, as little is known about the dynamic effects of M1-M2 polarization in the pathogenesis of DKD. Single cell RNA sequencing of immune cells showed a significant shift in macrophage subtypes in diabetic mice kidneys with an increase in both M1 and M2 macrophages and a shift towards M1-like macrophages at 7 months as compared to control mice [47]. Hence, integrating such data with relevant model improvements could be useful in the future in predicting GEC activation and structural changes in disease states that depend directly on macrophage phenotype and individual cell-level dynamics.

The model suggests potential strategies for disease intervention that can complement established methods once validated. Often, clinical biomarkers of kidney disease prediction and progression risk, such as serum creatinine and albuminuria, only show alterations relatively late in the disease process and, thus, may not be suitable for early disease diagnosis [98]. Therefore, the proposed mechanisms support our understanding of new disease biomarkers that are more closely related to histological changes in the early development and progression of DKD. When validated, they can potentially improve the early diagnosis and clinical management of the disease. The proposed mechanistic interactions based on diabetic mice data may also be relevant in predicting changes in fenestration dynamics in the clinical population, as a quantitatively similar effect on fenestrations in diabetic nephropathy patients was observed [10].

## Conclusion

In this study, we used a previously developed LBODEs model of protein-protein interactions for GECs *in vitro* and extended it to study the development and progression of DKD *in vivo*. The LBODEs network model predicted the effects of high glucose and inflammation on macrophage phenotypic changes, GEC activation, and signaling dysregulation in diabetic mice. Further, the extended LBODEs model related the signaling dysregulation with histological changes in GEC fenestrations, and mechanistic relationships were calibrated using *in vivo* mice data. Through *in silico* targeted inhibition, we identified the effective response time and confirmed mechanisms of action and effect on fenestrations through glucose control, species or pathway inhibition, or known chemical agents tested in other fenestrated endothelial cell types. We identified that disease intervention strategies besides glucose control are essential in regulating the complex downstream signals and pathways that control fenestrations in diseased GECs. Inhibition of network species, such as Rho/Rock, VEGF-A, VEGFR2, VEGF-A (mRNA), and NFκB, restored fenestration number and diameter, and pMLC restored fenestration diameter. Reducing the interaction strengths between Rho/Rock and pMLC and between NFκB, VEGF-A (mRNA), and VEGF-A were most effective in restoring fenestration diameter. The novel logic-based network model helped to quantify the cross talk between macrophages and GECs in the early through late stages of DKD in mice. The proposed mechanisms support our understanding of new disease biomarkers or pathways more closely related to histological changes in DKD development and progression. The proposed model could be integrated in the future with more complex models for other glomerular cells to better predict other aspects of disease progression and identify early biomarkers for DKD, enhancing clinical management and intervention strategies.

## Supporting information

S1 Appendix Supplementary equations (Eqs (S1)-(S34)), supplementary tables (Tables A-E), and supplementary figures (Figs A-M). (PDF)
