# Supplementary material for: Multi-cellular network model predicts alterations in glomerular endothelial structure in diabetic kidney disease
Source: PLoS Comput Biol. 2025 Oct 23;21(10):e1013598. doi: 10.1371/journal.pcbi.1013598 (PMC12561992; doi:10.1371/journal.pcbi.1013598)
Supplement: S1 Appendix — (PDF) [file pcbi.1013598.s001.pdf]

# Supporting information for Multi-cellular network model predicts alterations in glomerular endothelial structure in diabetic kidney disease

Krutika Patidar and Ashlee N. Ford Versypt

## Supplementary equations

In addition to the equations reported in the main text, the following LBODEs in Eqs (S1)-(S34) also govern network interactions for species $i = 2:35$.

$$\frac{d\mathrm{Actin_s}}{dt} = \frac{y_{\max_{\mathrm{Actin_s}}} f_{\mathrm{act}_{46}}(\mathrm{pMLC}) - \mathrm{Actin_s}}{\tau_{\mathrm{Actin_s}}} \tag{S1}$$

$$\frac{d\mathrm{AGE}}{dt} = \frac{y_{\max_{\mathrm{AGE}}} f_{\mathrm{act}_{3}}(\mathrm{GLU}) - \mathrm{AGE}}{\tau_{\mathrm{AGE}}} \tag{S2}$$

$$\frac{d\mathrm{VEGFR1}}{dt} = \frac{y_{\max_{\mathrm{VEGFR1}}} f_{\mathrm{act}_{18}}(\mathrm{VEGF\text{-}A}) - \mathrm{VEGFR1}}{\tau_{\mathrm{VEGFR1}}} \tag{S3}$$

$$\frac{d\mathrm{VEGFR2}}{dt} = \frac{y_{\max_{\mathrm{VEGFR2}}} f_{\mathrm{act}_{19}}(\mathrm{VEGF\text{-}A}) - \mathrm{VEGFR2}}{\tau_{\mathrm{VEGFR2}}} \tag{S4}$$

$$\frac{d\mathrm{VEGF\text{-}A_{mRNA}}}{dt} = \frac{y_{\max_{\mathrm{VEGF\text{-}A_{mRNA}}}} f_{\mathrm{act}_{15}}(\mathrm{NF\kappa B}) - \mathrm{VEGF\text{-}A_{mRNA}}}{\tau_{\mathrm{VEGF\text{-}A_{mRNA}}}} \tag{S5}$$

$$\frac{d\mathrm{RAGE_{ec}}}{dt} = \frac{y_{\max_{\mathrm{RAGE_{ec}}}} f_{\mathrm{act}_{20}}(\mathrm{AGE}) - \mathrm{RAGE_{ec}}}{\tau_{\mathrm{RAGE_{ec}}}} \tag{S6}$$

$$\frac{d\mathrm{RAGE}}{dt} = \frac{y_{\max_{\mathrm{RAGE}}} f_{\mathrm{act}_{4}}(\mathrm{AGE}) - \mathrm{RAGE}}{\tau_{\mathrm{RAGE}}} \tag{S7}$$

$$\frac{d\mathrm{IL\text{-}1R}}{dt} = \frac{y_{\max_{\mathrm{IL\text{-}1R}}} f_{\mathrm{act}_{2}}(\mathrm{IL\text{-}1\beta}) - \mathrm{IL\text{-}1R}}{\tau_{\mathrm{IL\text{-}1R}}} \tag{S8}$$

$$\frac{d\mathrm{NADPH}}{dt} = \frac{y_{\max_{\mathrm{NADPH}}} f_{\mathrm{act}_{5}}(\mathrm{RAGE}) - \mathrm{NADPH}}{\tau_{\mathrm{NADPH}}} \tag{S9}$$

$$\frac{d\mathrm{NADPH_{ec}}}{dt} = \frac{y_{\max_{\mathrm{NADPH_{ec}}}} f_{\mathrm{act}_{21}}(\mathrm{RAGE_{ec}}) - \mathrm{NADPH_{ec}}}{\tau_{\mathrm{NADPH_{ec}}}} \tag{S10}$$

$$\frac{d\mathrm{ROS_{ec}}}{dt} = \frac{y_{\max_{\mathrm{ROS_{ec}}}} OR(f_{\mathrm{act}_{33}}(\mathrm{eNOS}), f_{\mathrm{act}_{24}}(\mathrm{NADPH_{ec}})) - \mathrm{ROS_{ec}}}{\tau_{\mathrm{ROS_{ec}}}} \tag{S11}$$

$$\frac{d\mathrm{ROS}}{dt} = \frac{y_{\max_{\mathrm{ROS}}} OR\left(f_{\mathrm{act}_{10}}(\mathrm{PI3K}), f_{\mathrm{act}_{8}}(\mathrm{NADPH})\right) - \mathrm{ROS}}{\tau_{\mathrm{ROS}}} \tag{S12}$$

$$\frac{d\mathrm{PI3K}}{dt} = \frac{y_{\max_{\mathrm{PI3K}}} f_{\mathrm{act}_{7}}(\mathrm{IL} - 1\mathrm{R}) - \mathrm{PI3K}}{\tau_{\mathrm{PI3K}}} \tag{S13}$$

$$\frac{d\mathrm{AKT}}{dt} = \frac{y_{\max_{\mathrm{AKT}}} f_{\mathrm{act}_{9}}(\mathrm{PI3K}) - \mathrm{AKT}}{\tau_{\mathrm{AKT}}} \tag{S14}$$

$$\frac{d\mathrm{PI3K_{ec}}}{dt} = \frac{y_{\max_{\mathrm{PI3K_{ec}}}} OR(f_{\mathrm{act}_{23}}(\mathrm{VEGFR1}), f_{\mathrm{act}_{22}}(\mathrm{VEGFR2})) - \mathrm{PI3K_{ec}}}{\tau_{\mathrm{PI3K_{ec}}}} \tag{S15}$$

$$\frac{d\mathrm{AKT_{ec}}}{dt} = \frac{y_{\max_{\mathrm{AKT_{ec}}}} f_{\mathrm{act}_{25}}(\mathrm{PI3K_{ec}}) - \mathrm{AKT_{ec}}}{\tau_{\mathrm{AKT_{ec}}}} \tag{S16}$$

$$\frac{d\mathrm{NF\kappa B_{ec}}}{dt} = \frac{y_{\max_{\mathrm{NF\kappa B_{ec}}}} OR(f_{\mathrm{act}_{28}}(\mathrm{PLC\text{-}\gamma}), f_{\mathrm{act}_{29}}(\mathrm{ROS_{ec}})) - \mathrm{NF\kappa B_{ec}}}{\tau_{\mathrm{NF\kappa B_{ec}}}} \tag{S17}$$

$$\frac{d\mathrm{NF\kappa B}}{dt} = \frac{y_{\max_{\mathrm{NF\kappa B}}} OR(AND(f_{\mathrm{act}_{6}}(\mathrm{IL\text{-}1R}), f_{\mathrm{act}_{6}}(\mathrm{ROS})), f_{\mathrm{act}_{12}}(\mathrm{AKT})) - \mathrm{NF\kappa B}}{\tau_{\mathrm{NF\kappa B}}} \tag{S18}$$

$$\frac{d\mathrm{NO}}{dt} = \frac{y_{\max_{\mathrm{NO}}} OR(f_{\mathrm{act}_{32}}(\mathrm{eNOS}), f_{\mathrm{act}_{38}}(\mathrm{Ca})) - \mathrm{NO}}{\tau_{\mathrm{NO}}} \tag{S19}$$

$$\frac{d\mathrm{ONOO}}{dt} = \frac{y_{\max_{\mathrm{ONOO}}} AND(f_{\mathrm{act}_{34}}(\mathrm{NO}), f_{\mathrm{act}_{34}}(\mathrm{ROS_{ec}})) - \mathrm{ONOO}}{\tau_{\mathrm{ONOO}}} \tag{S20}$$

$$\frac{d\mathrm{eNOS}}{dt} = \frac{y_{\max_{\mathrm{eNOS}}} f_{\mathrm{act}_{26}}(\mathrm{AKT_{ec}}) - \mathrm{eNOS}}{\tau_{\mathrm{eNOS}}} \tag{S21}$$

$$\frac{d\mathrm{IL\text{-}6}}{dt} = \frac{y_{\max_{\mathrm{IL\text{-}6}}} OR(f_{\mathrm{act}_{13}}(\mathrm{NF\kappa B}), f_{\mathrm{act}_{30}}(\mathrm{NF\kappa B_{ec}})) - \mathrm{IL\text{-}6}}{\tau_{\mathrm{IL\text{-}6}}} \tag{S22}$$

$$\frac{d\mathrm{TNF\text{-}\alpha}}{dt} = \frac{y_{\max_{\mathrm{TNF\text{-}\alpha}}} OR(f_{\mathrm{act}_{14}}(\mathrm{NF\kappa B}), f_{\mathrm{act}_{11}}(\mathrm{NF\kappa B_{ec}})) - \mathrm{TNF\text{-}\alpha}}{\tau_{\mathrm{TNF\text{-}\alpha}}} \tag{S23}$$

$$\frac{d\mathrm{IL\text{-}\beta}}{dt} = \frac{y_{\max_{\mathrm{IL\text{-}1\beta}}} OR(f_{\mathrm{act}_{17}}(\mathrm{NF\kappa B}), f_{\mathrm{act}_{31}}(\mathrm{NF\kappa B_{ec}})) - \mathrm{IL\text{-}1\beta}}{\tau_{\mathrm{IL\text{-}1\beta}}} \tag{S24}$$

$$\frac{d\mathrm{PLC\text{-}\gamma}}{dt} = \frac{y_{\max_{\mathrm{PLC\text{-}\gamma}}} f_{\mathrm{act}_{27}}(\mathrm{VEGFR1}) - \mathrm{PLC\text{-}\gamma}}{\tau_{\mathrm{PLC\text{-}\gamma}}} \tag{S25}$$

$$\frac{d\mathrm{VEGF\text{-}A}}{dt} = \frac{y_{\max_{\mathrm{VEGF\text{-}A}}} f_{\mathrm{act}_{16}}(\mathrm{VEGF\text{-}A_{mRNA}}) - \mathrm{VEGF\text{-}A}}{\tau_{\mathrm{VEGF\text{-}A}}} \tag{S26}$$

$$\frac{d\mathrm{pJunction}}{dt} = \frac{y_{\max_{\mathrm{pJunction}}} f_{\mathrm{act}_{37}}(\mathrm{Ca}) - \mathrm{pJunction}}{\tau_{\mathrm{pJunction}}} \tag{S27}$$

$$\frac{d\mathrm{Ca}}{dt} = \frac{y_{\max_{\mathrm{Ca}}} OR(f_{\mathrm{inhib}_{35}}(\mathrm{NO}), f_{\mathrm{act}_{36}}(\mathrm{PLC\text{-}\gamma})) - \mathrm{Ca}}{\tau_{\mathrm{Ca}}} \tag{S28}$$

$$\frac{d\mathrm{Actin_r}}{dt} = \frac{y_{\max_{\mathrm{Actin_r}}} f_{\mathrm{act}_{47}}(\mathrm{MLC}) - \mathrm{Actin_r}}{\tau_{\mathrm{Actin_r}}} \tag{S29}$$

$$\frac{d\mathrm{RhoRock}}{dt} = \frac{y_{\max_{\mathrm{RhoRock}}} f_{\mathrm{act}_{43}}(\mathrm{VEGFR2}) - \mathrm{RhoRock}}{\tau_{\mathrm{RhoRock}}} \tag{S30}$$

$$\frac{d\mathrm{MLCK}}{dt} = \frac{y_{\max_{\mathrm{MLCK}}} OR(f_{\mathrm{act}_{40}}(\mathrm{Ca}), AND(f_{\mathrm{inhib}_{39}}(\mathrm{NO}), f_{\mathrm{act}_{39}}(\mathrm{ROS_{ec}}))) - \mathrm{MLCK}}{\tau_{\mathrm{MLCK}}} \tag{S31}$$

$$\frac{d\mathrm{pMLC}}{dt} = \frac{y_{\max_{\mathrm{pMLC}}} OR(f_{\mathrm{act}_{41}}(\mathrm{RhoRock}), AND(f_{\mathrm{act}_{45}}(\mathrm{MLC}), f_{\mathrm{act}_{45}}(\mathrm{MLCK})) - \mathrm{pMLC}}{\tau_{\mathrm{pMLC}}} \tag{S32}$$

$$\frac{d\mathrm{MLCP}}{dt} = \frac{y_{\max_{\mathrm{MLCP}}} f_{\mathrm{inhib}_{44}}(\mathrm{RhoRock}) - \mathrm{MLCP}}{\tau_{\mathrm{MLCP}}} \tag{S33}$$

$$\frac{d\mathrm{MLC}}{dt} = \frac{y_{\max_{\mathrm{MLC}}} AND(f_{\mathrm{act}_{42}}(\mathrm{pMLC}), f_{\mathrm{act}_{42}}(\mathrm{MLCP})) - \mathrm{MLC}}{\tau_{\mathrm{MLC}}} \tag{S34}$$

## Supplementary tables

**Table A. Chemical species abbreviations and definitions used in the extended network (Fig 1).**

| Abbreviation | Definition |
|---|---|
| $\mathrm{Actin_s}$ | Stressed actin fibers |
| $\mathrm{Actin_s}$ | Relaxed actin fibers |
| AGE | advanced glycation end product |
| AKT | serine/threonine-specific protein kinases |
| Ca | calcium |
| eNOS | endothelial nitric oxide synthase |
| GLU | glucose |
| IL | interleukin |
| MLCK | myosin light chain kinase |
| MLCP | myosin light chain phosphatase |
| NADPH | nicotinamide adenine dinucleotide phosphate |
| NF$\kappa$B | nuclear factor kappa B |
| NO | nitric oxide |
| ONOO | peroxynitrite |
| PI3K | phosphoinositide 3-kinases |
| pJunction | phosphorylated junction protein |
| PLC-$\gamma$ | phospholipase C gamma |
| RAGE | receptor of advanced glycation end product |
| Rock | Rho-associated protein kinase |
| ROS | reactive oxygen species |
| TLR | toll-like receptor |
| TNF-$\alpha$ | tumor necrosis factor-alpha |
| VEGF | vascular endothelial growth factor |
| VEGFR | vascular endothelial growth factor receptor |

**Table B. List of species parameter $\tau_i$ for the LBODEs model organized by species index $i$.** The time constant ($\tau_i$) values in units of hours are set based on realistic timescales for activation of the respective proteins [1]. The default value of $\tau_i$ is 1 hour. The default values of the other species parameters are initial value $y_{0_i} = 0$ and maximal value $y_{\max_i} = 1$ for all species unless stated otherwise. Number and Diameter represent fenestration number and fenestration diameter, respectively.

| ID | Species | $\tau_i$ (hour) | $y_{\max_i}$ | $y_{0_i}$ |
|---|---|---|---|---|
| 1 | GLU | 1 | 1 | 0 |
| 2 | $\text{Actin}_\text{s}$ | 1 | 1 | 0 |
| 3 | AGE | 1 | 1 | 0 |
| 4 | VEGFR1 | 0.35 | 1 | 0 |
| 5 | VEGFR2 | 0.35 | 1 | 0 |
| 6 | $\text{VEGF-A}_\text{mRNA}$ | 88 | 1 | 0 |
| 7 | $\text{RAGE}_\text{ec}$ | 0.35 | 1 | 0 |
| 8 | RAGE | 0.35 | 1 | 0 |
| 9 | IL-1R | 0.35 | 1 | 0 |
| 10 | NADPH | 1 | 1 | 0 |
| 11 | $\text{NADPH}_\text{ec}$ | 1 | 1 | 0 |
| 12 | $\text{ROS}_\text{ec}$ | 1 | 1 | 0 |
| 13 | ROS | 1 | 1 | 0 |
| 14 | PI3K | 1 | 1 | 0 |
| 15 | AKT | 1 | 1 | 0 |
| 16 | $\text{PI3K}_\text{ec}$ | 1 | 1 | 0 |
| 17 | $\text{AKT}_\text{ec}$ | 1 | 1 | 0 |
| 18 | $\text{NF}\kappa\text{B}_\text{ec}$ | 0.055 | 1 | 0 |
| 19 | $\text{NF}\kappa\text{B}$ | 0.055 | 1 | 0 |
| 20 | NO | 1 | 1 | 0 |
| 21 | ONOO | 1 | 1 | 0 |
| 22 | eNOS | 1 | 1 | 0 |
| 23 | IL-6 | 90 | 1 | 0 |
| 24 | TNF-$\alpha$ | 90 | 1 | 0 |
| 25 | IL-1$\beta$ | 90 | 1 | 0 |
| 26 | PLC-$\gamma$ | 1 | 1 | 0 |
| 27 | VEGF-A | 1.13 | 1 | 0 |
| 28 | pJunction | 1 | 1 | 0 |
| 29 | Ca | 1 | 1 | 0 |
| 30 | $\text{Actin}_\text{r}$ | 1 | 1 | 0 |
| 31 | RhoRock | 1 | 1 | 0 |
| 32 | MLCK | 1 | 1 | 0 |
| 33 | pMLC | 400 | 1 | 0 |
| 34 | MLCP | 1 | 1 | 0 |
| 35 | MLC | 1 | 1 | 0 |
| 36 | Number | – | – | 6.3 |
| 37 | Diameter | – | – | 47.91 nm |

**Table C. List of optimal reaction parameters for the LBODEs model organized by reaction index $j$.** Reaction weight ($W_j$), Hill coefficient ($n_j$), and half-maximal effect ($EC_{50_j}$) are shown for respective reaction rules or interactions. Reaction rule A ⇒ C denotes input A activates output C. Reaction rule !A ⇒ C denotes input A inhibits output C. Reaction rule A & B ⇒ C denotes the *AND* logic operator between input A and input B to give output C. Default values are $W_j = 1$, $n_j = 1.4$, and $EC_{50_j} = 0.5$. (–) indicates that the respective parameters do not apply to the reaction.

| ID | Reaction | $W_j$ | $n_j$ | $EC_{50_j}$ |
|---|---|---|---|---|
| 1 | ⇒ GLU | – | – | – |
| 2 | IL-1β ⇒ IL-1R | 1 | 1.4 | 0.5 |
| 3 | GLU ⇒ AGE | 1 | 1.45 | 0.5 |
| 4 | AGE ⇒ RAGE | 1 | 2.71 | 0.474 |
| 5 | RAGE ⇒ NADPH | 0.944 | 2.7 | 0.470 |
| 6 | IL-1R & ROS ⇒ NFκB | 1 | 1.4 | 0.5 |
| 7 | IL-1R ⇒ PI3K | 1 | 1.4 | 0.5 |
| 8 | NADPH ⇒ ROS | 0.943 | 2.64 | 0.545 |
| 9 | PI3K ⇒ AKT | 1 | 2.7 | 0.5 |
| 10 | PI3K ⇒ ROS | 0.950 | 2.7 | 0.419 |
| 11 | $NF\kappa B_{ec}$ ⇒ TNF-α | 0.999 | 3.96 | 0.840 |
| 12 | AKT ⇒ NFκB | 1 | 2.7 | 0.5 |
| 13 | NFκB ⇒ IL-6 | 0.950 | 2.7 | 0.420 |
| 14 | NFκB ⇒ TNF-α | 0.950 | 2.7 | 0.419 |
| 15 | NFκB ⇒ VEGF-$A_{mRNA}$ | 0.95 | 2.7 | 0.422 |
| 16 | VEGF-$A_{mRNA}$ ⇒ VEGF-A | 0.949 | 2.77 | 0.595 |
| 17 | NFκB ⇒ IL-1β | 0.950 | 2.7 | 0.421 |
| 18 | VEGF-A ⇒ VEGFR1 | 1 | 2.71 | 0.5 |
| 19 | VEGF-A ⇒ VEGFR2 | 1 | 2.72 | 0.5 |
| 20 | AGE ⇒ $RAGE_{ec}$ | 1 | 1.56 | 0.839 |
| 21 | $RAGE_{ec}$ ⇒ $NADPH_{ec}$ | 0.938 | 1.6 | 0.839 |
| 22 | VEGFR2 ⇒ $PI3K_{ec}$ | 1 | 2.73 | 0.5 |
| 23 | VEGFR1 ⇒ $PI3K_{ec}$ | 1 | 2.72 | 0.5 |
| 24 | $NADPH_{ec}$ ⇒ $ROS_{ec}$ | 0.946 | 3.91 | 0.838 |
| 25 | $PI3K_{ec}$ ⇒ $AKT_{ec}$ | 1 | 2.93 | 0.688 |
| 26 | $AKT_{ec}$ ⇒ eNOS | 1 | 3.11 | 0.576 |
| 27 | VEGFR1 ⇒ PLC | 1 | 1.4 | 0.5 |
| 28 | PLC ⇒ $NFKB_{ec}$ | 1 | 1.4 | 0.5 |
| 29 | $ROS_{ec}$ ⇒ $NFKB_{ec}$ | 1 | 2.7 | 0.010 |
| 30 | $NFKB_{ec}$ ⇒ IL-6 | 0.986 | 2.7 | 0.471 |
| 31 | $NFKB_{ec}$ ⇒ IL-1β | 0.962 | 2.7 | 0.391 |
| 32 | eNOS ⇒ NO | 1 | 1.46 | 0.157 |
| 33 | eNOS ⇒ $ROS_{ec}$ | 1 | 3.66 | 0.833 |
| 34 | $ROS_{ec}$ & NO ⇒ ONOO | 1 | 1.4 | 0.5 |
| 35 | !NO ⇒ Ca | 1 | 2.68 | 0.5 |
| 36 | PLC-γ ⇒ Ca | 1 | 1.4 | 0.5 |
| 37 | Ca ⇒ pJunction | 1 | 1.4 | 0.5 |
| 38 | Ca ⇒ NO | 1 | 1.4 | 0.5 |
| 39 | !NO & $ROS_{ec}$ ⇒ MLCK | 1 | 1.4 | 0.5 |
| 40 | Ca ⇒ MLCK | 1 | 1.4 | 0.5 |
| 41 | RhoRock ⇒ pMLC | 1 | 1.4 | 0.5 |
| 42 | pMLC & MLCP ⇒ MLC | 1 | 1.4 | 0.5 |
| 43 | VEGFR2 ⇒ RhoRock | 1 | 1.4 | 0.5 |
| 44 | !RhoRock ⇒ MLCP | 1 | 1.4 | 0.5 |
| 45 | MLC & MLCK ⇒ pMLC | 1 | 1.4 | 0.5 |
| 46 | pMLC ⇒ $Actin_s$ | 1 | 1.4 | 0.5 |
| 47 | MLC ⇒ $Actin_r$ | 1 | 1.4 | 0.5 |

**Table D. Experimentally observed effects (promotion ↑ or inhibition ↓) of chemical agents on respective targeted species in liver sinusoidal endothelial cells (LSECs) [2].** LSEC porosity is the number of fenestrations per unit area. LSEC diameter is the fenestration diameter.

| Chemical agent | Target | Effect on LSEC porosity | Effect on LSEC diameter |
|---|---|---|---|
| KN93 | Ca | ↓ | no change |
| ML-7 | MLCK | ↓ | ↑ |
| Y27632 | Rock | ↑ | ↓ |
| Calyculin A | MLCP | ↓ | ↑ |
| Cytochalasin B | $Actin_s$ | ↑ | ↑ |

**Table E. Mean and standard deviation (SD) of predicted fenestration number and diameter after *in silico* intervention with chemical agents via complete protein knockdown (Fig 10) of the targets listed in Table D.**

| Chemical agent | Fenestration Diameter Mean (SD) | Fenestration Number Mean (SD) |
|---|---|---|
| No treatment | 75.11 ($1.1 \times 10^{-2}$) | 4.491 ($3.7 \times 10^{-6}$) |
| KN93 | 75.09 ($1.2 \times 10^{-2}$) | 4.491 ($4.1 \times 10^{-6}$) |
| ML7 | 75.09 ($1.4 \times 10^{-2}$) | 4.491 ($4.9 \times 10^{-6}$) |
| Y27632 | 47.91 ($7.1 \times 10^{-15}$) | 6.300 (0.0) |
| Calyculin A | 75.08 ($1.3 \times 10^{-2}$) | 4.054 ($4.5 \times 10^{-5}$) |
| Cytochalasin B | 75.11 ($1.1 \times 10^{-2}$) | 6.696 ($2.2 \times 10^{-4}$) |

# Supplementary figures

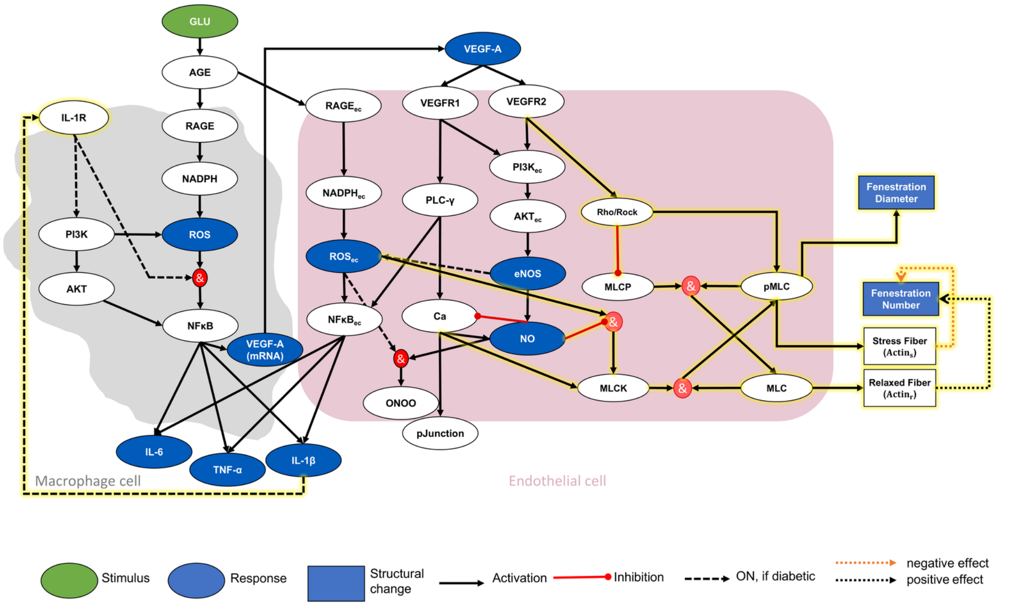


**Fig A. Multi-cellular protein interaction network of *in vivo* cross talk between macrophages and glomerular endothelial cells.** The protein interaction network between macrophages (gray area) and glomerular endothelial cells (pink area) is stimulated with static or dynamic glucose. Yellow highlighted nodes and interaction edges are additions in the extended model compared to those in the previous model [3]. For more details on the node descriptions, refer to the main text.

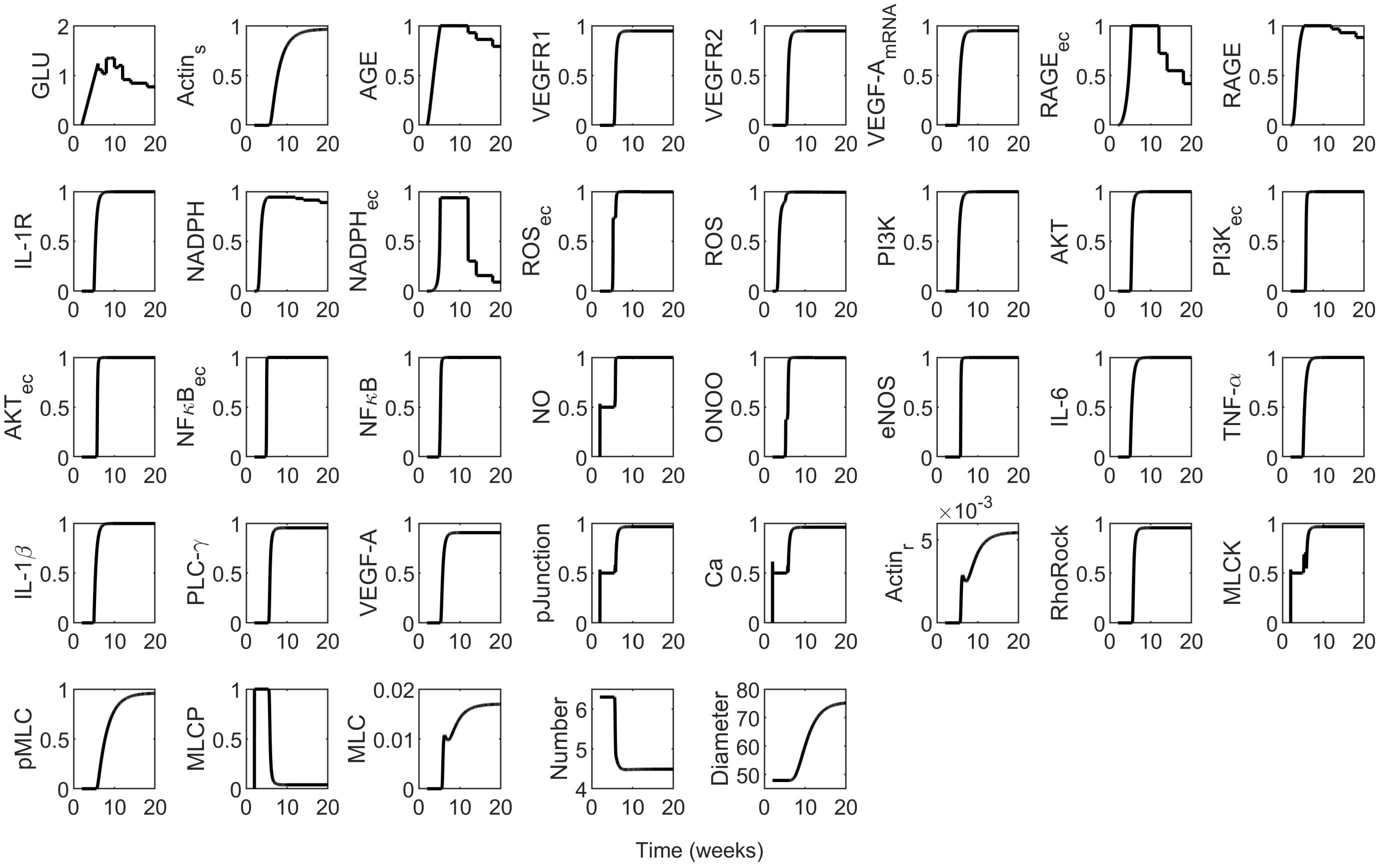


**Fig B. Predicted dynamics of the species in the multi-cellular protein interaction network (Fig 1) simulated using the single mean glucose trajectory $G(t)$ (Fig 2) as input.**

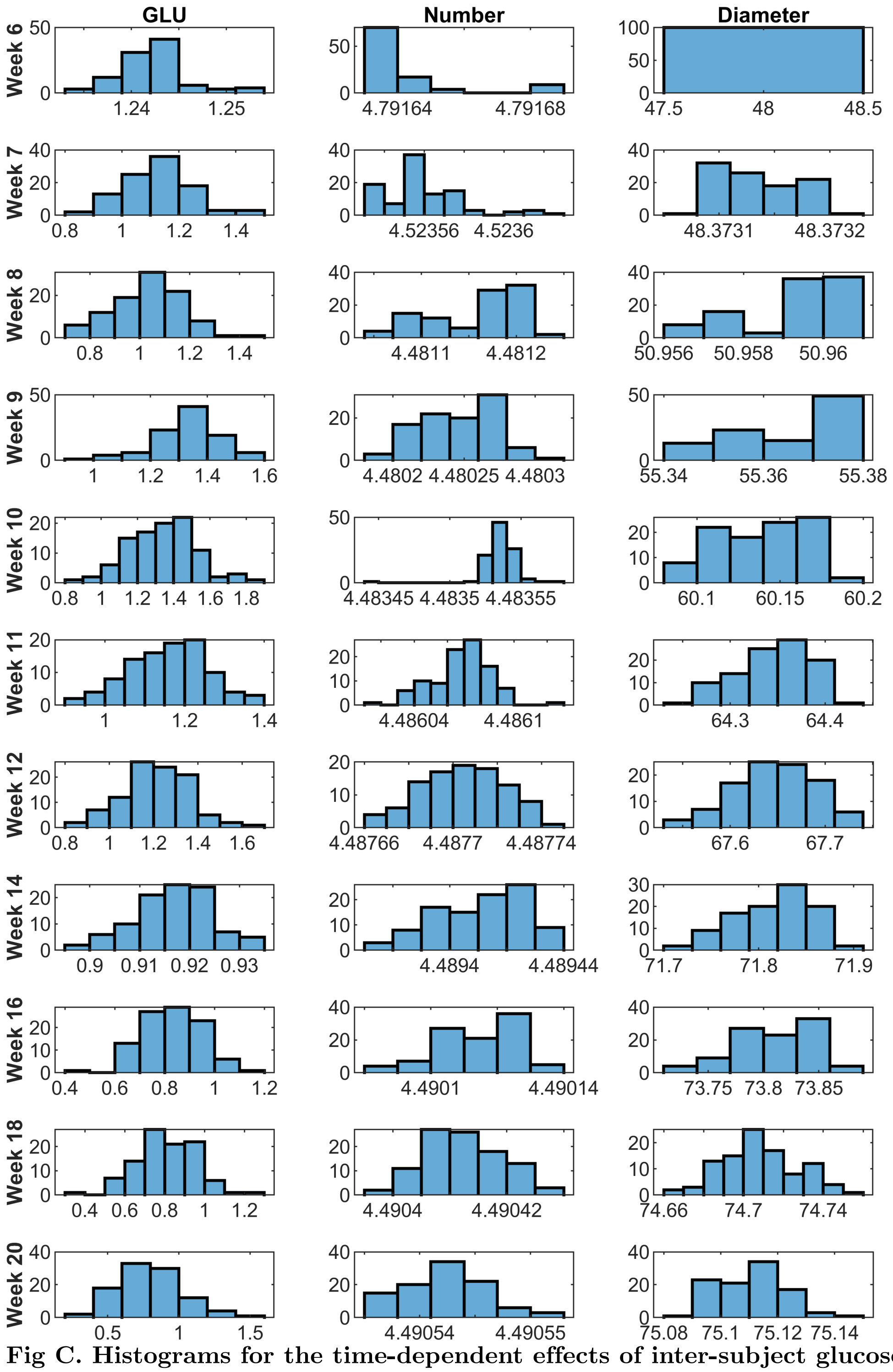


**Fig C. Histograms for the time-dependent effects of inter-subject glucose variability using the 100 $G(t)$ trajectories (Fig 3) as input for the virtual mouse population ($n = 100$).** Glucose was sampled $n$ times in each time interval for 6–20 weeks by drawing from a normal distribution with mean and standard deviation from the data at the start of the corresponding time interval (labeled on the rows). (Column 1) Histograms of the sampled GLU distributions in each time interval. Histograms of predicted values of fenestration number (Column 2) and fenestration diameter (Column 3).

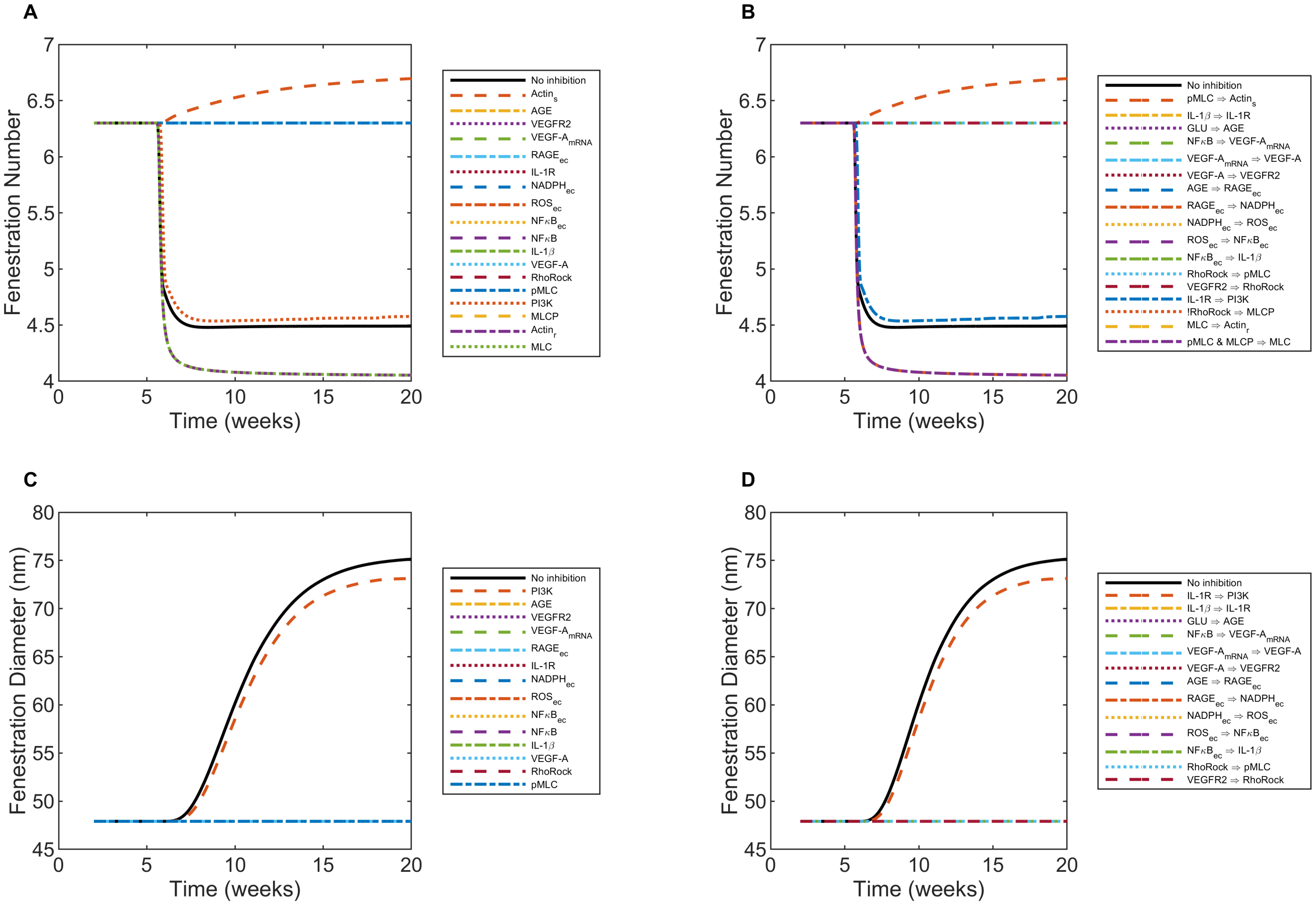


**Fig D. Structural effects over time as a result of the sensitivity analysis for sensitive parameters.** At the initial time, each of the species parameters ($y_{\mathrm{max_i}}$) and reaction parameters ($W_j$) was reduced one-at-a-time by 100%. A, B: Fenestration number output for perturbed parameters for sensitive A: species and B: reactions. C, D: Fenestration diameter output for perturbed parameters for sensitive C: species and D: reactions. Black curves (labeled as "No inhibition") on each panel serve as the controls and show the structural effects without inhibition.

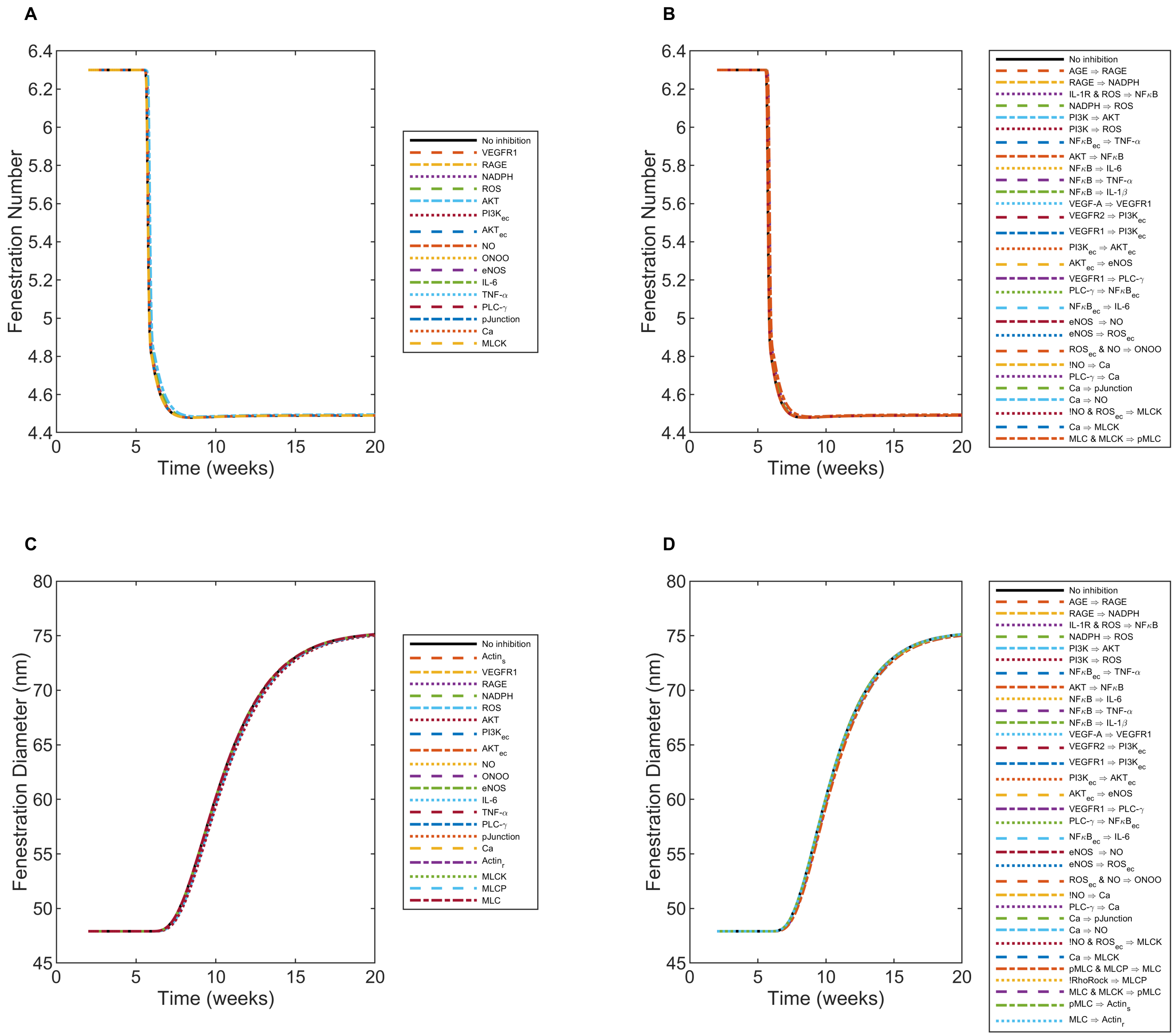


**Fig E. Structural effects over time as a result of the sensitivity analysis for non-sensitive parameters.** At the initial time, each of the species parameters ($y_{\max_i}$) and reaction parameters ($W_j$) was reduced one-at-a-time by 100%. A, B: Fenestration number output for perturbed parameters for non-sensitive A: species and B: reactions. C, D: Fenestration diameter output for perturbed parameters for non-sensitive C: species and D: reactions. Black curves (labeled as "No inhibition") on each panel serve as the controls and show the structural effects without inhibition.

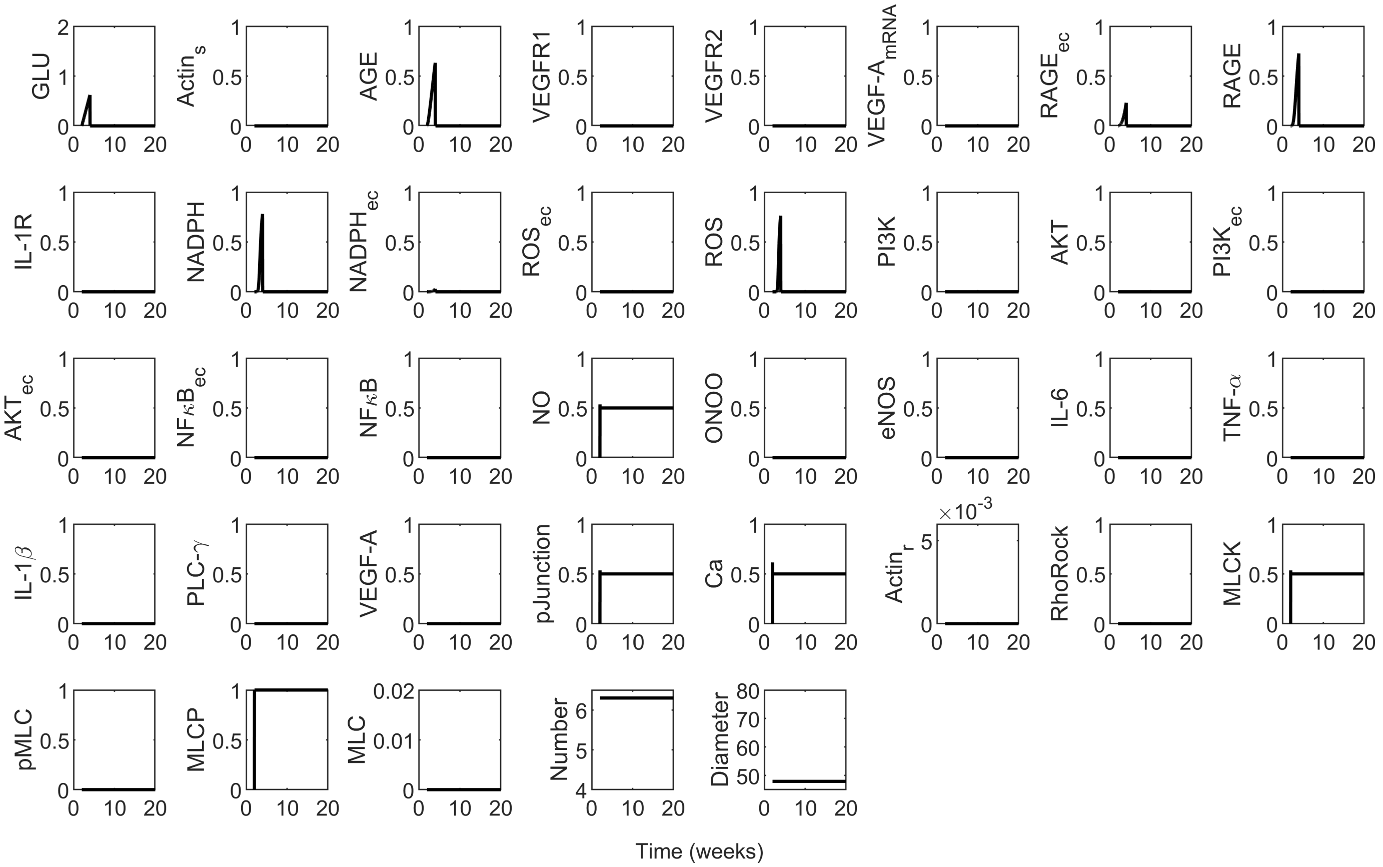


**Fig F. Predicted dynamics of the species in the multi-cellular protein interaction network (Fig 1) simulated using the single mean glucose trajectory $G(t)$ (Fig 2) as input.** Glucose control ($W'_{\mathrm{GLU}}(t) = 0$) was applied at 4 weeks.

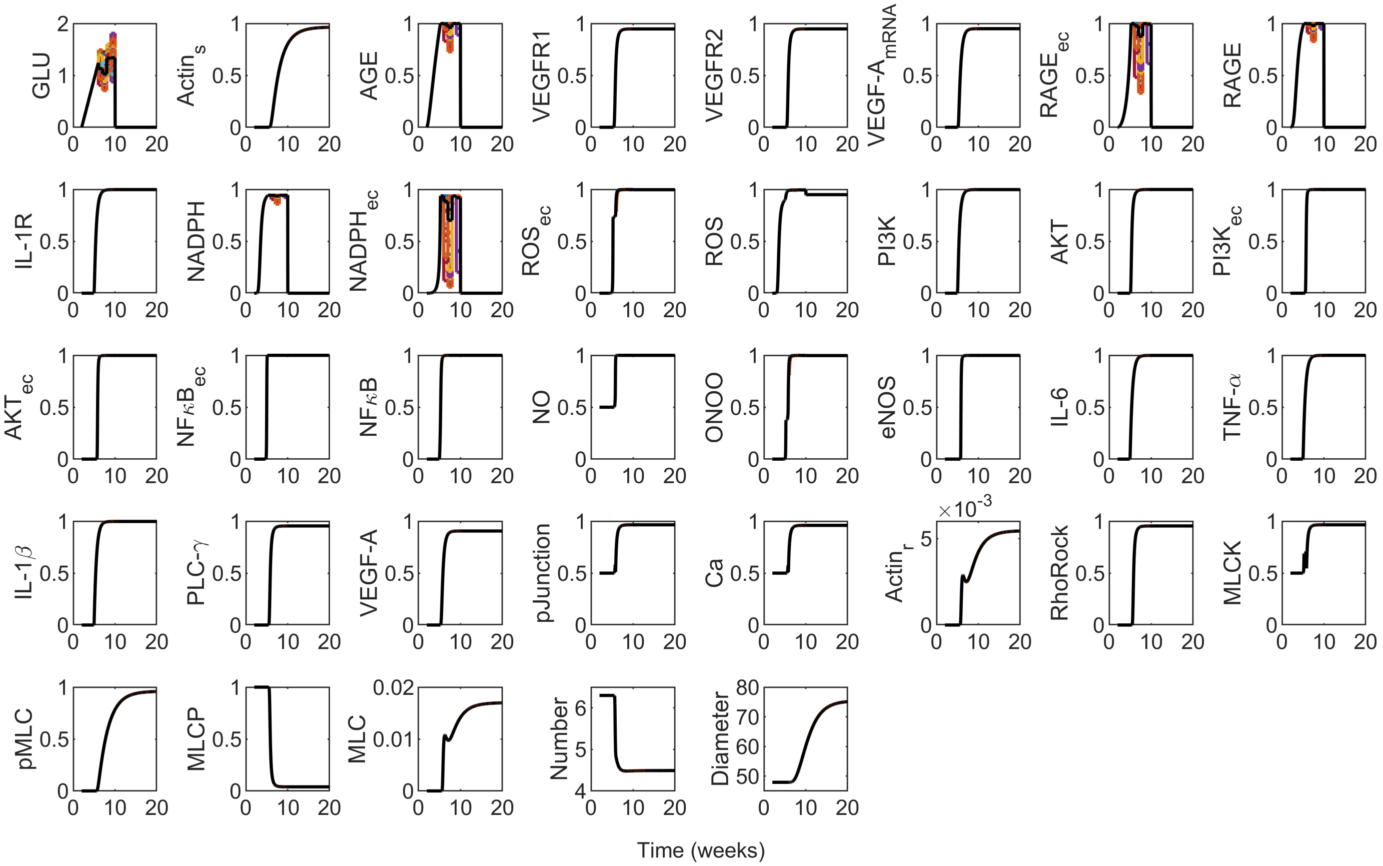


**Fig G. Predicted dynamics of the species in the multi-cellular protein interaction network (Fig 1) simulated using the 100 $G(t)$ trajectories (Fig 3) for the virtual mouse population as input.** Glucose control ($W'_{\mathrm{GLU}}(t) = 0$) was applied at 10 weeks. Note that the means of the outputs from the 100 input glucose trajectories are shown in black, while the dynamic outputs for individuals cycle through MATLAB's default color order.

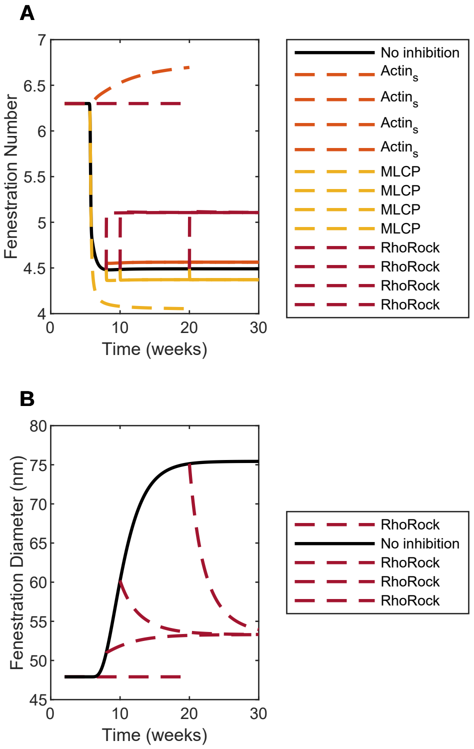


**Fig H. Structural effects over time as a result of the *in silico* intervention with chemical agents targeted sensitive parameters (Table D).** At different times (the initial time of 2 weeks and 8, 10, and 20 weeks), each of the targeted species parameters ($y_{\mathrm{max_i}}$) was reduced one-at-a-time by 100% for the initial time or by 50% for all other times. A: Fenestration number output for perturbed parameters for sensitive target species. B: Fenestration diameter output for perturbed parameters for sensitive target species. Black curves (labeled as "No inhibition") on each panel serve as the controls and show the structural effects without inhibition. Actin$_s$ inhibition corresponds to cytochalasin B treatment. Rock inhibition corresponds to Y27632 treatment. MLCP inhibition corresponds to calyculin A treatment. For all treatment targets that do not appear in the legend, the targets are non-sensitive and their respective responses do not differ from the No inhibition responses.

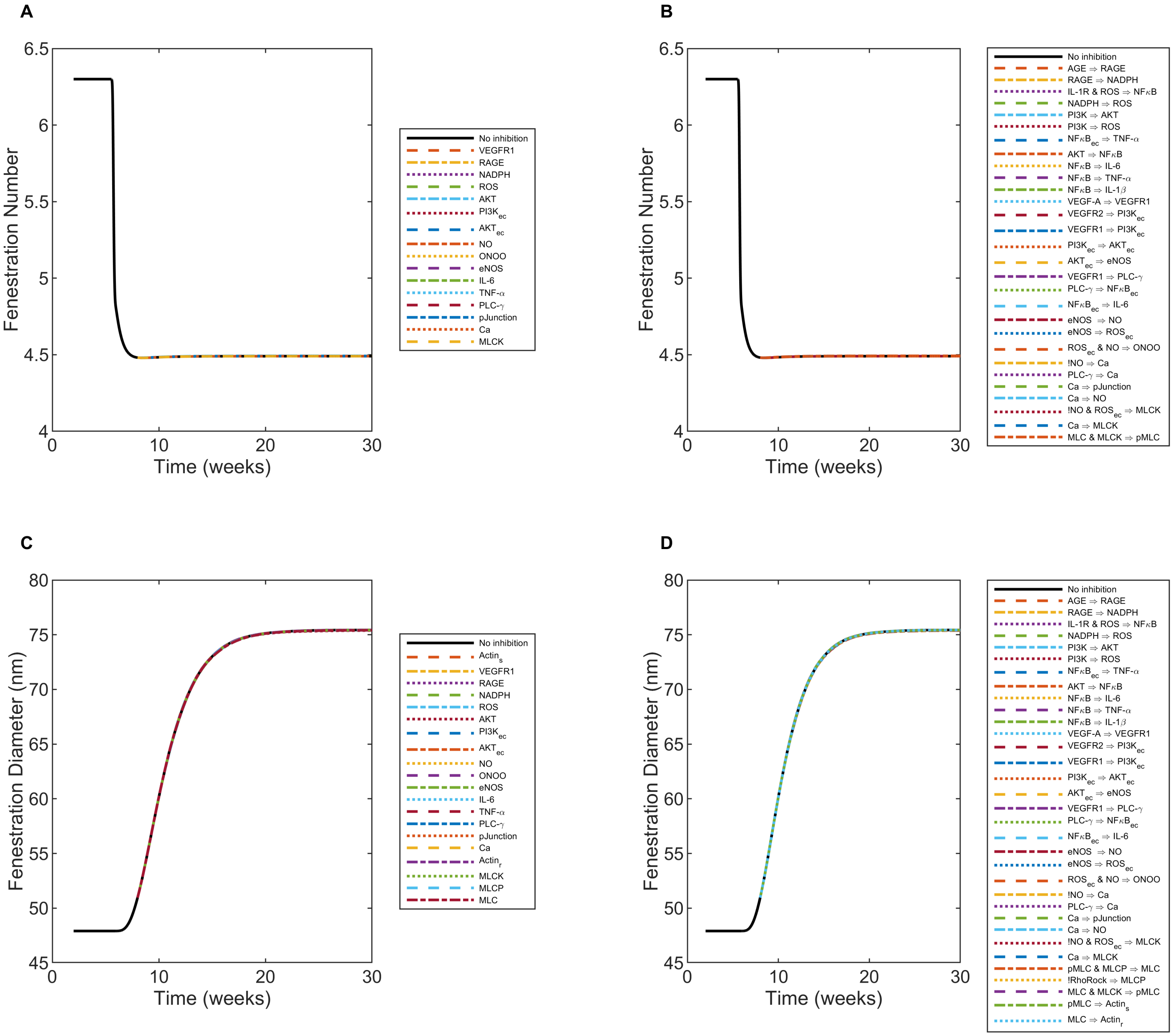


**Fig I. Structural effects over time as a result of the perturbation analysis for inhibiting non-sensitive parameters at 8 weeks.** After starting at their optimal values, each of the species parameters ($y_{\mathrm{max_i}}$) and reaction parameters ($W_j$) was reduced one-at-a-time by 50%. A, B: Fenestration number output for perturbed parameters for non-sensitive A: species and B: reactions. C, D: Fenestration diameter output for perturbed parameters for non-sensitive C: species and D: reactions. Black curves (labeled as "No inhibition") on each panel serve as the controls and show the structural effects without inhibition.

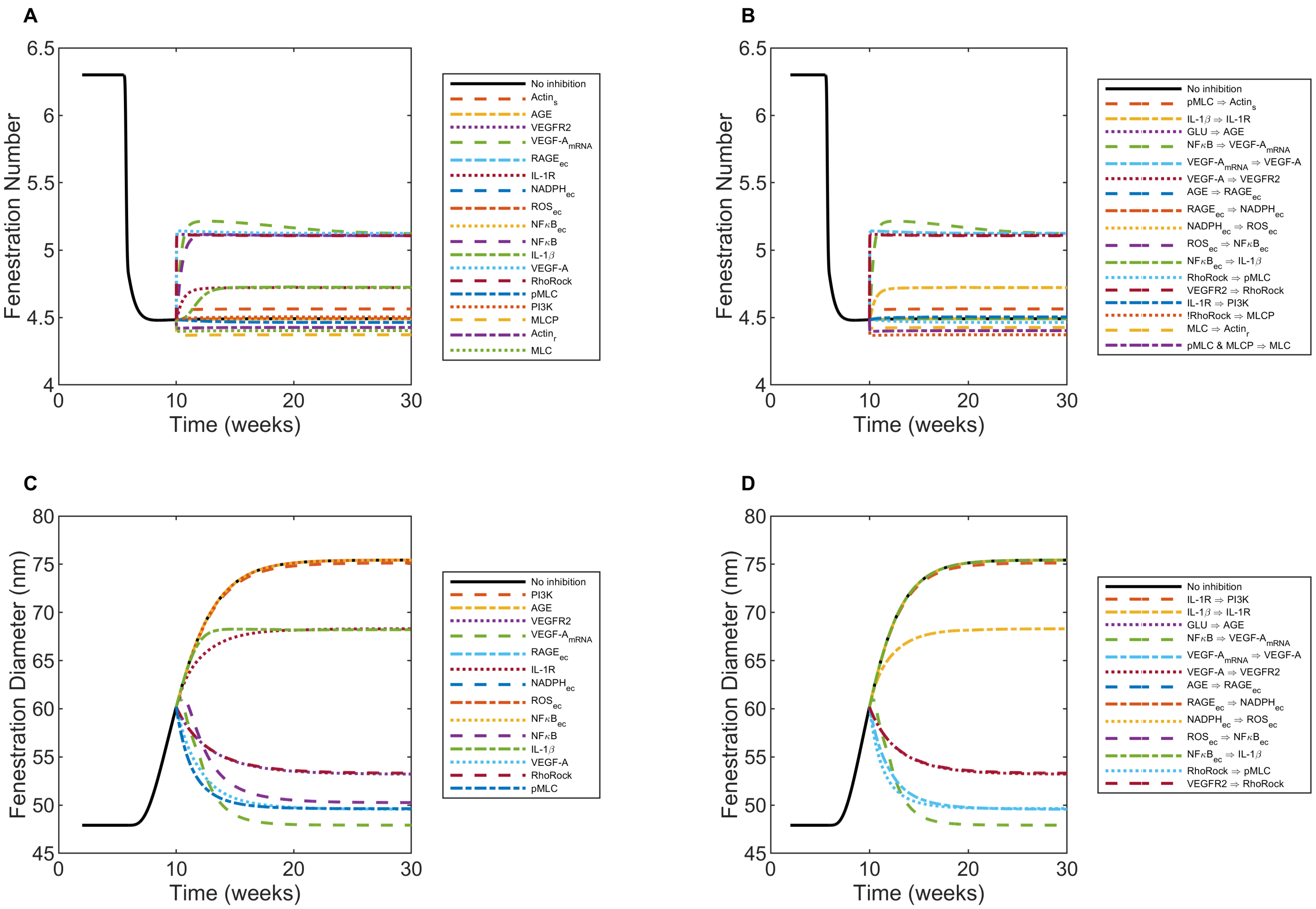


**Fig J. Structural effects over time as a result of the perturbation analysis for inhibiting sensitive parameters at 10 weeks.** After starting at their optimal values, each of the species parameters ($y_{\max_i}$) and reaction parameters ($W_j$) was reduced one-at-a-time by 50%. A, B: Fenestration number output for perturbed parameters for sensitive A: species and B: reactions. C, D: Fenestration diameter output for perturbed parameters for sensitive C: species and D: reactions. Black curves (labeled as "No inhibition") on each panel serve as the controls and show the structural effects without inhibition.

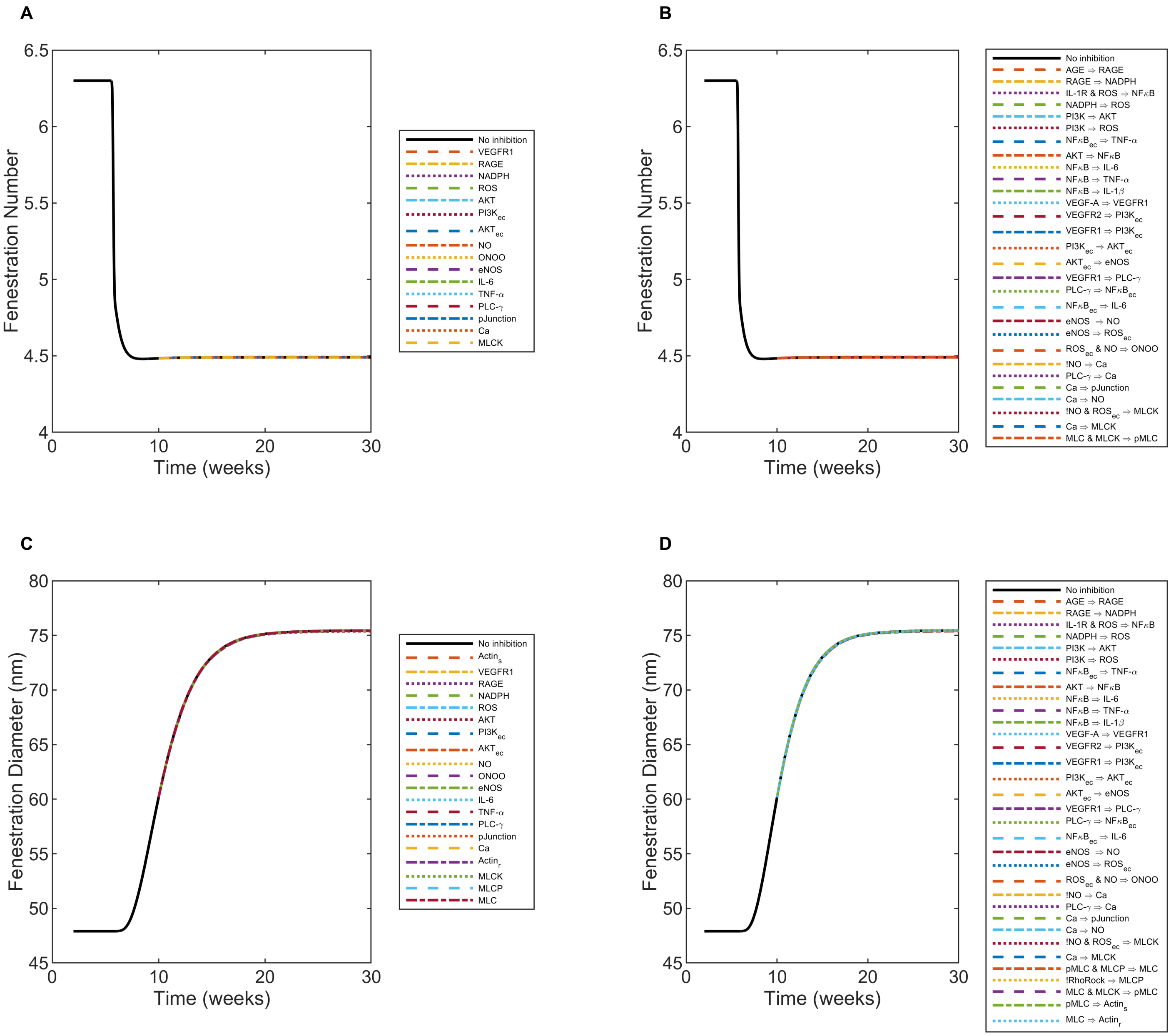


**Fig K. Structural effects over time as a result of the perturbation analysis for inhibiting non-sensitive parameters at 10 weeks.** After starting at their optimal values, each of the species parameters ($y_{\mathrm{max_i}}$) and reaction parameters ($W_j$) was reduced one-at-a-time by 50%. A, B: Fenestration number output for perturbed parameters for non-sensitive A: species and B: reactions. C, D: Fenestration diameter output for perturbed parameters for non-sensitive C: species and D: reactions. Black curves (labeled as "No inhibition") on each panel serve as the controls and show the structural effects without inhibition.

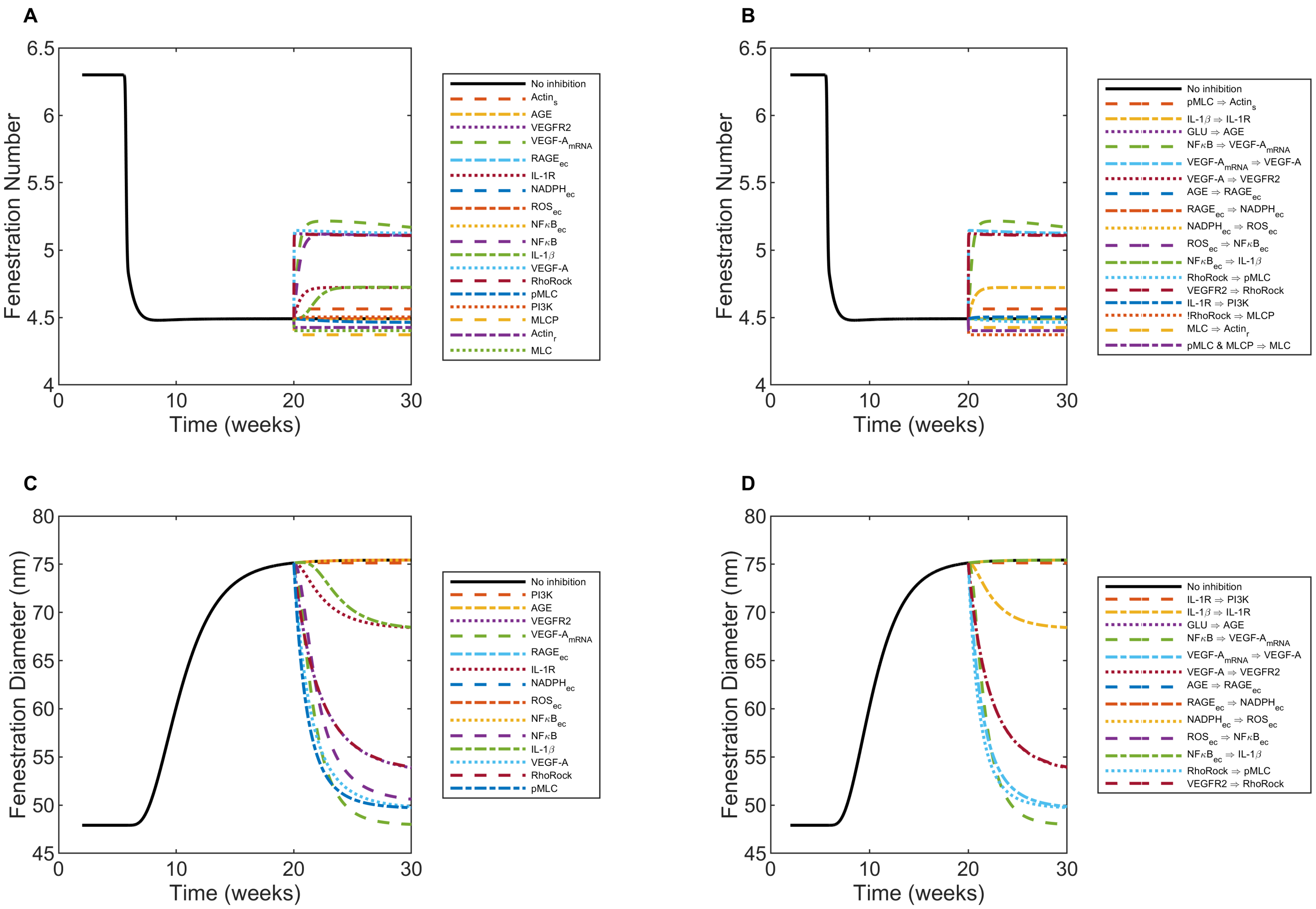


**Fig L. Structural effects over time as a result of the perturbation analysis for inhibiting sensitive parameters at 20 weeks.** After starting at their optimal values, each of the species parameters ($y_{\max_i}$) and reaction parameters ($W_j$) was reduced one-at-a-time by 50%. A, B: Fenestration number output for perturbed parameters for sensitive A: species and B: reactions. C, D: Fenestration diameter output for perturbed parameters for sensitive C: species and D: reactions. Black curves (labeled as "No inhibition") on each panel serve as the controls and show the structural effects without inhibition.

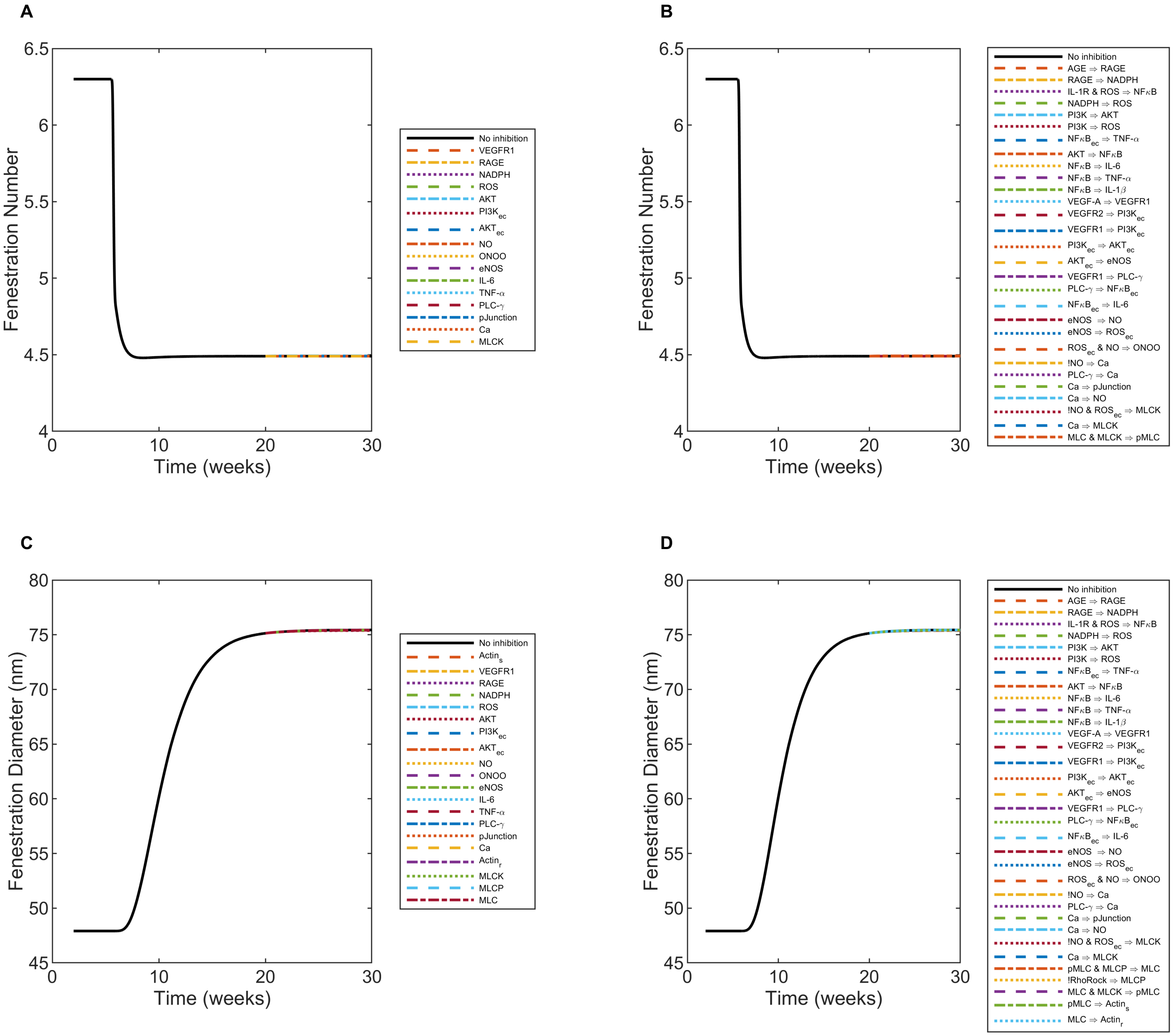


**Fig M. Structural effects over time as a result of the perturbation analysis for inhibiting non-sensitive parameters at 20 weeks.** After starting at their optimal values, each of the species parameters ($y_{\mathrm{max_i}}$) and reaction parameters ($W_j$) was reduced one-at-a-time by 50%. A, B: Fenestration number output for perturbed parameters for non-sensitive A: species and B: reactions. C, D: Fenestration diameter output for perturbed parameters for non-sensitive C: species and D: reactions. Black curves (labeled as "No inhibition") on each panel serve as the controls and show the structural effects without inhibition.

# References

1. Klinke II DJ, Finley SD. Timescale analysis of rule-based biochemical reaction networks. Biotechnol Prog. 2012;28:33-44. doi:10.1002/btpr.704.

2. Zapotoczny B, Szafranska K, Lekka M, Ahluwalia BS, McCourt P. Tuning of liver sieve: the interplay between actin and myosin regulatory light chain regulates fenestration size and number in murine liver sinusoidal endothelial cells. Int J Mol Sci. 2022;23:9850. doi:10.3390/ijms23179850.

3. Patidar K, Ford Versypt AN. Logic-based modeling of inflammatory macrophage cross talk with glomerular endothelial cells in diabetic kidney disease. Am J Physiol Renal Physiol. 2025;329(1):F202-24. doi:10.1152/ajprenal.00362.2024.
